# The Effect of the First UK COVID-19 Lockdown on Users of the Drink Less App: Interrupted Time Series Analysis of Sociodemographic Characteristics, Engagement, and Alcohol Reduction

**DOI:** 10.2196/42320

**Published:** 2022-11-10

**Authors:** Melissa Oldham, Olga Perski, Gemma Loebenberg, Jamie Brown, Claire Garnett

**Affiliations:** 1 Department of Behavioural Science and Health University College London London United Kingdom

**Keywords:** alcohol reduction, COVID-19, digital intervention, smartphone app, United Kingdom, alcohol, app, Drink Less, engagement, users, lockdown, female

## Abstract

**Background:**

The first UK COVID-19 lockdown had a polarizing impact on drinking behavior and may have impacted engagement with digital interventions to reduce alcohol consumption.

**Objective:**

We examined the effect of lockdown on engagement, alcohol reduction, and the sociodemographic characteristics of users of the popular and widely available alcohol reduction app Drink Less.

**Methods:**

This was a natural experiment. The study period spanned 468 days between March 24, 2019, and July 3, 2020, with the introduction of UK lockdown measures beginning on March 24, 2020. Users were 18 years or older, based in the United Kingdom, and interested in drinking less. Interrupted time series analyses using generalized additive mixed models (GAMMs) were conducted for each outcome variable (ie, sociodemographic characteristics, app downloads and engagement levels, alcohol consumption, and extent of alcohol reduction) for existing (downloaded the app prelockdown) and new (downloaded the app during the lockdown) users of the app.

**Results:**

Among existing users of the Drink Less app, there were increases in the time spent on the app per day (B=0.01, *P*=.01), mean units of alcohol recorded per day (B>0.00 *P*=.02), and mean heavy drinking (>6 units) days (B>0.00, *P*=.02) during the lockdown. Previous declines in new app downloads plateaued during the lockdown (incidence rate ratio [IRR]=1.00, *P*=.18). Among new app users, there was an increase in the proportion of female users (B>0.00, *P*=.04) and those at risk of alcohol dependence (B>0.00, *P*=.01) and a decrease in the proportion of nonmanual workers (B>–0.00, *P*=.04). Among new app users, there were step increases in the mean number of alcohol units per day (B=20.12, *P*=.03), heavy-drinking days (B=1.38, *P*=.01), and the number of days the app was used (B=2.05, *P*=.02), alongside a step decrease in the percentage of available screens viewed (B=–0.03, *P*=.04), indicating users were using less of the intervention components within the app.

**Conclusions:**

Following the first UK lockdown, there was evidence of increases in engagement and alcohol consumption among new and existing users of the Drink Less app.

## Introduction

Alcohol consumption is a dose-dependent [1], leading risk factor for preventable cases of cancer [2] and is linked with many other chronic and acute conditions [3]. Restrictions introduced as a result of the COVID-19 pandemic impacted drinking behavior, with rises in increasing and higher-risk drinking (defined by standard cut-offs of ≥8 on the full Alcohol Use Disorders Identification Test [AUDIT]) [4-6] and heavy episodic drinking [6] in the United Kingdom. Reducing alcohol-related harms is a public health priority [7]. This study reports the possible impact of COVID-19 restrictions on app engagement, alcohol reduction, and the sociodemographic characteristics of existing and new users of the moderately popular alcohol reduction app *Drink Less* [8].

The first lockdown was introduced in the United Kingdom in response to the COVID-19 pandemic, in March 2020. Following initial government advice from March 16, 2020, to avoid group gatherings and to work from home, where possible, the first national lockdown was announced with behavioral restriction measures coming into force in the United Kingdom from the March 24, 2020, remaining in place until July 4, 2020 [9]. Two subsequent national lockdowns and other social distancing measures followed. During the lockdown, all nonessential stores and licensed premises were closed, social gathering was prohibited, and the opportunity to drink alcohol outside the home was limited. Although pubs, clubs, and bars were closed, people could still purchase alcohol for consumption at home and domestic alcohol expenditure increased in the first lockdown [10,11]. Evidence suggests that the first lockdown had a polarizing impact on drinking patterns [6,12], with 26% of drinkers drinking less and 26% drinking more [12] and people replacing on-trade consumption with increases in own-home drinking [13]. The prevalence of increasing and higher-risk drinking increased significantly, with 1.8 times greater odds during the lockdown relative to the prelockdown period [4].

In addition to leading to increased alcohol consumption among some groups, the first lockdown also led to an increase in self-reported alcohol reduction attempts by increasing and higher-risk drinkers (28.5% during the lockdown vs 15.3% prelockdown) [4]. Many nonemergency, in-person National Health Service (NHS) facilities were temporarily closed during the lockdown, which could have made it more difficult for those motivated to cut down to access advice from health care professionals or specialist support services. As the opportunity to engage with physical health services was limited during the lockdown, we might expect to see an increase in the use of digital interventions during this period.

Digital interventions, such as websites and apps, are convenient and low cost [14] and accessible to most people in the United Kingdom [15]. Despite this, alcohol reduction apps are less frequently used than more traditional support services, with only around 4% of drinkers using one when attempting to cut down between 2015 and 2018 [16]. The context in which a digital intervention is used affects engagement [17], and it is unclear how the lockdown affected engagement with alcohol reduction apps. Being furloughed from work or having limited socialization opportunities could have resulted in more new users downloading apps or existing app users having a greater opportunity to engage with them. This could be reflected in the frequency, amount, and depth of use among existing users. Alternatively, competing priorities, such as adjusting to working from home, childcare, or other caring responsibilities, may have negatively impacted the time spent using alcohol reduction apps. Although many apps are available, few are informed by evidence and theory [18,19]. Drink Less is a free app containing multiple intervention components informed by behavior change theory and evidence, including a drinking diary and goal setting, that aims to support users in reducing their alcohol consumption [20,21] and has over 70,000 unique users, with a 4.5 star rating in the App Store [8]. The full process of developing and refining Drink Less, along with the intervention content, is reported elsewhere [20,22].

Understanding how the initial lockdown affected engagement with digital alcohol interventions and subsequent alcohol reduction could inform the targeting of public health messaging and the provision of alcohol support in the future. It is also important to consider the consistency of these effects across the population. Drinkers who drink more heavily [23], are more deprived [24,25], and are younger [26,27] are more likely to experience alcohol-related harms and may also have been differentially affected by the lockdown [28-30]. For example, more deprived groups are more likely to have unstable incomes and greater financial concerns and may have been isolated in less comfortable, more crowded accommodation [31]. This likely impacted the motivation, opportunity, and capability to engage with digital interventions and reduce drinking. Before the lockdown, users of Drink Less tended to be of higher socioeconomic status (SES) and heavier drinkers [32]. As such, it is important to consider the characteristics of those engaging with digital interventions designed to support alcohol reduction and whether this changed in the lockdown in order to monitor the possibility of emerging or worsened inequalities.

The aims of this paper are threefold. First, to understand how the use of Drink Less and drinking behavior may have changed during the lockdown, we examined whether the lockdown affected engagement and recorded drinking behavior among existing Drink Less users (ie, those who downloaded the app prelockdown). Second, we examined whether the lockdown led to a change in new app downloads. Third, to understand how the lockdown may have impacted the characteristics of users downloading the Drink Less app, we analyzed whether the sociodemographic characteristics of users, engagement with the app, and drinking behaviors recorded in the app differed between new users downloading the app prelockdown and during the lockdown.

This study addresses the following 3 research questions (RQs):

RQ1: Was the first UK lockdown associated with an immediate change in existing users of the Drink Less app in terms of:

- The depth of use (percent of available screens viewed)
- The amount of use (mean time spent on the app)
- The frequency of use (number of sessions)
- The number of alcohol units recorded each day
- The number of alcohol-free days recorded each day
- The number of heavy-drinking days recorded each day

RQ2: Was the first UK lockdown associated with a change in the number of new Drink Less downloads per day?

RQ3: Was the first UK lockdown associated with an immediate change in new users of the Drink Less app in terms of:

- Sociodemographic and drinking characteristics at baseline and in the 28 days following app download
- The depth of use (percentage of available screens viewed)
- The amount of use (mean time spent on the app)
- The frequency of use (number of sessions and number of days used)
- The number of alcohol units recorded
- The number of alcohol-free days recorded
- The number of heavy-drinking days recorded

## Methods

### Design

This was a natural experiment without active recruitment.

### Intervention and Study Period

For all RQs, the interruption was conceptualized as the introduction of national lockdown measures in the United Kingdom on March 24, 2020. Due to differences in the way that the independent variables were operationalized (see the Analysis section), the time periods for RQ1, RQ2, and RQ3a differed from RQ3b-g. Specifically, for RQ1, each of the outcome variables was aggregated at a daily level by the number of active users in that week. For RQ2, the number of new downloads each day was attributed to the day of download. For RQ3a, baseline AUDIT scores and sociodemographic variables were measured once and attributed to the day of download. As such, data were collected for the 468 days between March 24, 2019, and July 3, 2020. This captured the period of time up until pubs in England reopened on July 4, 2020, which could have had a stepped effect on alcohol consumption. This period was divided into pre- (March 24, 2019-March 23, 2020; 366 days) and during-lockdown (March 24, 2020-July 3, 2020; 102 days) segments. For RQ3b-g, the depth, amount, and frequency of use, along with the number of alcohol units, alcohol-free days, and heavy-drinking days, recorded were aggregated over the 28-day period following app download by the number of users who downloaded the app on that day. Therefore, to limit potential confounding after pubs reopened, only respondents downloading the app a full 28 days prior to when pubs reopened (up to June 6, 2020) were included. Therefore, the study period for RQ3b-g was March 24, 2019-June 6, 2020 (441 days) and was divided into pre- (March 24, 2019-March 23, 2020; 366 days) and during-lockdown (March 24, 2020-June 6, 2020; 75 days) segments.

### Study Population

The sample was UK users who downloaded the Drink Less app from Apple App Store, where it is freely available. To be eligible for inclusion, users had to be aged 18 years or older, based in the United Kingdom, interested in drinking less (specified when downloading the app), and have agreed to the privacy policy and terms and conditions within the app, as well as completing the AUDIT. For RQ1, existing users were defined as all those who downloaded the app between March 24, 2019, and the March 23, 2020 (prelockdown). RQ1 focused on existing, regular users, defined as use of the app at least once a week for a minimum of 4 weeks. For RQ2 and RQ3a, new users were defined as those who downloaded the app between March 24, 2020, and July 3, 2020, with no limits on regularity of use for new or existing users. Finally, for RQ3b-g, new users were defined as those who downloaded the app between March 24, 2020, and June 6, 2020, with no limits on regularity of use for new or existing users.

### Measures

#### Sociodemographic and Drinking Characteristics

Three sociodemographic characteristics were measured at download. These were age (in years, continuous), sex (percentage female), and employment type (percentage nonmanual). The AUDIT was asked of all users providing both an AUDIT score (continuous) and the percentage of increasing and higher-risk drinkers (AUDIT score>=8).

#### Number of Downloads

The number of new app downloads each day was recorded.

#### Engagement Indicators

Three indicators of user engagement were derived from screen view records for each user: (1) number of sessions (where a new session is defined as a new screen view after 30 minutes of inactivity), (2) time spent on the app in minutes, and (3) percentage of available screens viewed. For RQ1, these measures were aggregated at a daily level across active users and attributed to the day of engagement. For RQ3b-g, each measure was aggregated over the 28-day period following app download for each user and was attributed to the date of download. Due to the differences in aggregation, the number of days used was also included as a measure of engagement for RQ3.

#### Drinking Measures

In the app, users were prompted to fill in a daily drinking calendar, where they either marked days as “alcohol free” or entered any alcoholic drinks they drank that day. Three drinking variables were calculated: (1) number of alcohol units (UK unit=10 mL of ethanol) consumed (aggregated daily), (2) number of alcohol-free days, and (3) number of heavy-drinking days (defined as >6 alcohol units). As described before, these measures were operationalized differently for RQ1 and RQ3.

### Analysis

All analyses were conducted in R Studio. The engagement measures were derived using Pandas, a Python framework, within a Jupyter Notebook, an open source web application.

Models 1a-1f (for RQ1) examined whether the lockdown was associated with the percentage of available screens viewed, the mean time spent on the app, the number of sessions on the app, the number of alcohol units, the number of alcohol-free days, and the number of heavy-drinking days among existing, regular users of the Drink Less app.

Model 2 (for RQ2) examined whether the lockdown was associated with a change in the number of new daily downloads of the Drink Less app.

Models 3a-3e (for RQ3a) examined whether the lockdown was associated with changes in age, the proportion of female users, the proportion of nonmanual users, the proportion of users who were at risk of alcohol dependence, and the AUDIT scores among new downloaders of the Drink Less app.

Models 3f-3l (for RQ3b-g) examined whether there were changes in the percentage of available screens viewed, the mean time spent on the app, the number of sessions on the app, the number of days the app was used, the number of units, the number of alcohol-free days, and the number of heavy-drinking days recorded among new users following the first UK lockdown.

To estimate the associations between the lockdown and each of the outcomes, we conducted separate interrupted time series analyses using generalized additive mixed models (GAMMs). Analyses were conducted at the daily aggregated level while controlling for day of the week and month of the year. Smoothing “splines” were fitted in order to account for seasonal nonlinear variations in, for example, drinking behavior. To account for differences in the trends prelockdown and during the lockdown, the regression models included terms for the baseline level for each outcome prelockdown, the trend in the prelockdown period, the level change in the outcome immediately after the lockdown, and the trend in the during-lockdown period.

Plots of the autocorrelation functions (ACFs) and partial autocorrelation functions (PACFs) were used to test for both autoregressive (AR) and moving average (MA) autocorrelation over time. The ACF plots were used to identify plausible values for AR and MA terms for the baseline model. Models with various plausible AR and MA terms were compared with our baseline model using the Akaike information criterion (AIC), where smaller values indicate a better model fit.

As little was known about how the trends in each of the outcome variables during the lockdown, secondary analyses assessed whether regression models with nonlinear trends (cubic and quadratic) provided a better fit to the data. Best-fitting models were selected with the AIC, and where appropriate, cubic and quadratic models were reported.

All continuous variables were normally distributed, but a negative binomial distribution was used for the number of new downloads (RQ2), as the outcome variable was operationalized as a discrete (rather than continuous) variable and overdispersion was present. Data and details for each model (ie, AR and MA terms, AICs) are available in the annotated R code and can be accessed through GitHub [33].

### Sensitivity Analyses

Preplanned sensitivity analyses (adjusting the date of the interruption to that on which social distancing measures were introduced, March 17, 2020, and controlling for potential confounders) were not conducted due to the complexity of the paper, the practical constraints associated with running additional analyses, and the robust model selection approach already taken.

### Ethical Considerations

Ethical approval was obtained from University College London’s Research Ethics Committee (CEHP/2016/556; CEHP/2020/579), and participants provided online consent to having their anonymous data used for scientific research purposes.

## Results

### Descriptive Statistics

Table 1 reports descriptive statistics for the outcome variables for each of the RQs.

**Table 1:** Descriptive statistics for the aggregated outcome variables of interest, stratified by period (prelockdown vs during the lockdown).

RQs^a^		Entire period (441 days)	Entire period (468 days)	Prelockdown (366 days)	During the lockdown (75 days)^b^	During the lockdown (102 days)
**RQ1, mean (SD)**						
	Mean percentage screens viewed	N/A^c^	4.66 (0.80)	4.80 (0.83)	N/A	4.16 (0.30)
	Mean time spent on app (minutes)	N/A	1.58 (0.72)	1.73 (0.73)	N/A	1.05 (0.25)
	Mean number of logins	N/A	0.95 (0.11)	0.97 (0.11)	N/A	0.89 (0.06)
	Mean alcohol units per day	N/A	3.29 (1.52)	3.38 (1.55)	N/A	2.98 (1.36)
	Mean heavy-drinking days	N/A	0.21 (0.10)	0.21 (0.10)	N/A	0.18 (0.09)
	Mean alcohol-free days	N/A	0.46 (0.16)	0.48 (0.16)	N/A	0.42 (0.16)
**RQ2, median (IQR)^d^**						
	Number of new downloads per day	N/A	14.50 (13.00)	18.00 (13.00)	N/A	7.5 (6.00)
**RQ3a, mean (SD)**						
	Gender (proportion female)	N/A	0.54 (0.16)	0.53 (0.15)	N/A	0.57 (0.21)
	Age (years)	N/A	44.28 (4.07)	44.16 (3.64)	N/A	44.73 (5.34)
	Employment type (proportion nonmanual)	N/A	0.71 (0.15)	0.71 (0.13)	N/A	0.69 (0.19)
	AUDIT score	N/A	16.47 (2.41)	16.40 (2.06)	N/A	16.71 (3.40)
	At risk of alcohol dependence (proportion at risk)	N/A	0.91 (0.09)	0.91 (0.08)	N/A	0.90 (0.11)
**RQ3b-e, mean (SD)**						
	Number of logins	15.18 (6.60)	N/A	14.80 (5.80)	17.05 (9.42)	N/A
	Number of days used	9.66 (3.41)	N/A	9.46 (2.96)	10.65 (4.97)	N/A
	Percentage screens viewed	0.31 (0.04)	N/A	0.30 (0.04)	0.31 (0.06)	N/A
	Time spent on app (minutes)	39.08 (21.64)	N/A	38.73 (20.28)	40.81 (27.51)	N/A
	Alcohol units	72.21 (36.65)	N/A	70.60 (31.51)	80.15 (55.10)	N/A
	Heavy-drinking days	4.44 (2.19)	N/A	4.35 (1.63)	4.90 (3.94)	N/A
	Alcohol-free days	10.70 (5.42)	N/A	10.74 (5.31)	10.48 (5.93)	N/A

^a^RQ: research question.

^b^Shorter during-lockdown period as outcome variables 3b-e were aggregated over 28 days rather than at a monthly level.

^c^N/A: not applicable.

^d^Median (IQR) presented here; a large variance could lead to a skewed mean.

### Association of the First UK Lockdown With Engagement With the Drink Less App Among Existing, Regular Users (RQ1)

There was an overall decline in the mean time spent on the app, the mean units recorded per day, and the mean heavy-drinking days recorded during the study period, with no step changes following the first COVID-19 lockdown. However, there was a change in slope during the lockdown, with a significant increase in the trajectory in the mean time spent on the app, the mean units recorded per day, and the mean heavy-drinking days recorded following the lockdown, though the magnitude of the change in these daily trends appeared small (Table 2).

There was an overall decline in the mean percentage of screens viewed and the mean number of sessions during the study period, with no step change following the first COVID-19 lockdown in the United Kingdom. The declining trend plateaued during the lockdown, with no significant trend in the mean percentage of screens viewed or the mean number of sessions (Table 2).

**Figure 1:**
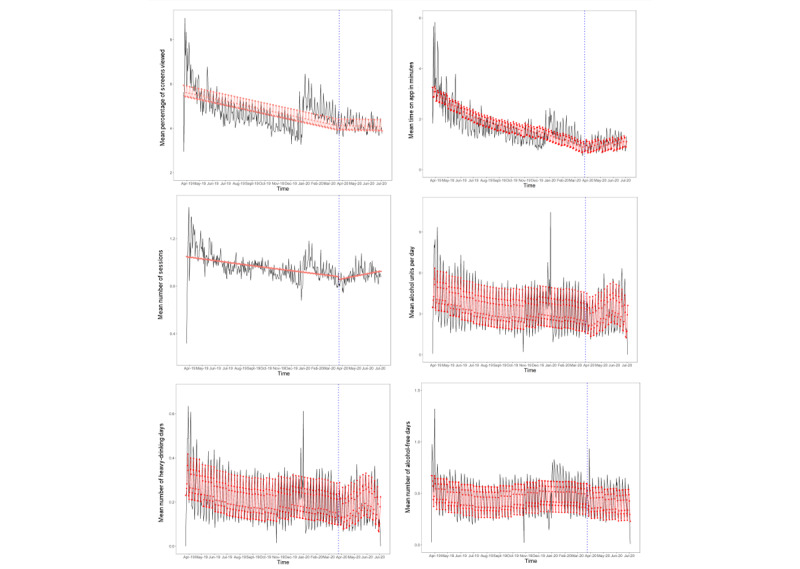
Engagement indicators among existing, regular users of the Drink Less app over the study period (RQ1a-f). The red line indicates fitted values, the gray area indicates the 95% CI, and the dashed blue line indicates the interruption (ie, the first national UK lockdown). RQ: research question. Higher-resolution version of this figure is available in Multimedia Appendix 1.

There was no significant trend in the mean alcohol-free days over the study period and no step change or change in slope following introduction of the first lockdown in the United Kingdom (Table 2 and Figure 1).

**Table 2:** Results of the best-fitting model for each outcome variable for RQ1^a^ (N=468 days, range 9-598 users per day).

Outcome variables		B (95% CI)		*P* value
**Mean percentage screens viewed^b^, linear model**				
	Trend	–0.0042 (–0.0074 to –0.0010)	.01	
	Level	0.0204 (–0.5541 to 0.5949)	.95	
	Slope	0.0038 (–0.0105 to 0.0181)	.60	
**Mean time spent on app^a^, linear model**				
	Trend	–0.0061 (–0.0081 to –0.0041)	.00	
	Level	0.0604 (–0.3230 to 0.4438)	.76	
	Slope	0.0118 (0.0027-0.0209)	.01	
**Mean number of sessions^b^, linear model**				
	Trend	–0.0005 (–0.0007 to –0.0003)	.00	
	Level	–0.0247 (–0.1083 to 0.0589)	.56	
	Slope	0.0012 (–0.0001 to 0.0025)	.09	
**Mean alcohol units per day^b^, cubic model**				
	Trend	–0.0049 (–0.0066 to –0.0032)	.00	
	Level	0.0351 (–0.9218 to 0.9920)	.94	
	Slope	–0.0297 (–0.1065 to 0.0471)	.45	
	Slope^2^	0.0016 (–0.0001 to 0.0033)	.07	
	Slope^3^	0.0000 (0.0000-0.0000)	.02	
**Mean heavy-drinking days^b^, cubic model**				
	Trend	–0.0004 (–0.0005 to –0.0003)	.00	
	Level	0.0106 (–0.0498 to 0.0710)	.73	
	Slope	–0.0019 (–0.0068 to 0.0030)	.44	
	Slope^2^	0.0001 (0.0000-0.0002)	.06	
	Slope^3^	0.0000 (0.0000-0.0000)	.02	
**Mean alcohol-free days^b^, linear model**				
	Trend	–0.0002 (–0.0004 to 0.0000)	.10	
	Level	–0.0183 (–0.1101 to 0.0735)	.70	
	Slope	0.0005 (–0.0009 to 0.0019)	.44	

^a^RQ: research question.

^b^Adjusted for month of the year (cubic spline), day of the week (cubic spline), and autocorrelation.

### Effect of the First UK Lockdown on the Number of Drink Less Downloads er Day (RQ2)

**Figure 2:**
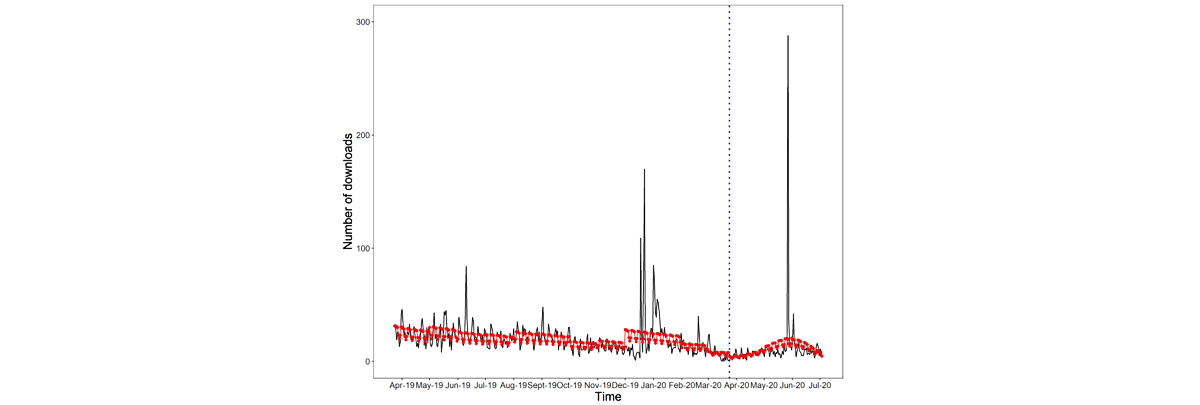
Number of new Drink Less downloads per day over the study period (RQ2). The red line indicates fitted values, the gray area indicates the 95% CI, and the dashed blue line indicates the interruption. RQ: research question.

There was an overall declining trend in the number of downloads per day over the full study period, with no step change detected following the first COVID-19 lockdown in the United Kingdom. However, the declining trend plateaued during the lockdown, with no significant trend in downloads per day (Table 3 and Figure 2).

**Table 3:** Results of the best-fitting model for the number of downloads (N=468 days; range 0-288 downloads per day).

Number of downloads^a^, cubic model	Incidence rate ratio (95% CI)	*P* value
Trend	0.9962 (0.9945-0.9979)	.00
Level	0.5365 (0.2332-1.2342)	.14
Slope	1.0161 (0.9495-1.0875)	.64
Slope^2^	1.0006 (0.9991-1.0021)	.45
Slope^3^	1.0000 (1.0000-1.0000)	.18

^a^Adjusted for month of the year (cubic spline), day of the week (cubic spline), and autocorrelation.

### Effect of the First UK Lockdown on Sociodemographic and Drinking Characteristics Among New Users of the Drink Less App (RQ3a)

**Figure 3:**
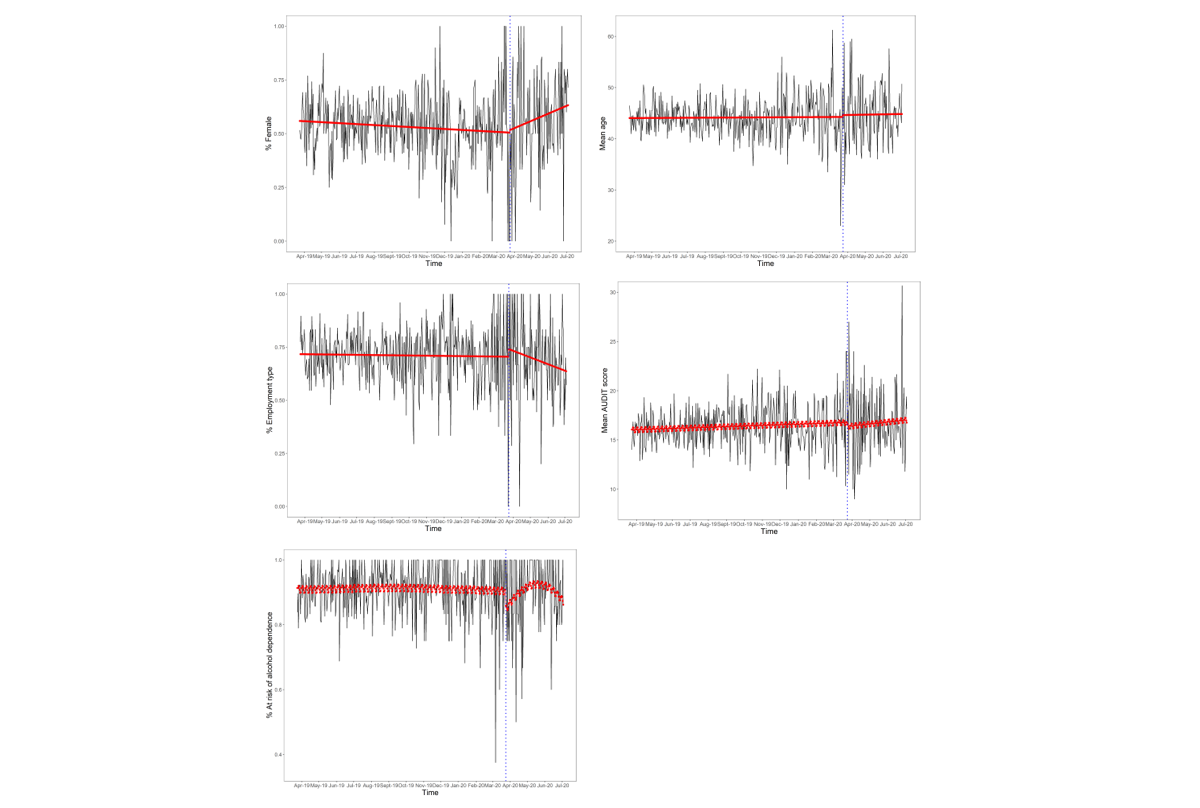
Sociodemographic and drinking characteristics of new users of the Drink Less app over the study period (RQ3a). The red line indicates fitted values, the gray area indicates the 95% CI, and the dashed blue line indicates interruption. AUDIT: Alcohol Use Disorders Identification Test; RQ: research question. Higher-resolution version of this figure is available in Multimedia Appendix 1.

There was no significant overall trend detected in the proportion of female users over the study period or the proportion of nonmanual (vs manual) workers, with no step change following the lockdown. However, there was a change in slope following the introduction of the first UK lockdown to a significant upward trajectory in the proportion of female users and a significant negative trajectory in the proportion of nonmanual workers (Table 4 and Figure 3).

There was no significant overall trend in the proportion of new users who were at risk of alcohol dependence over the study period. However, there was a step decrease during the lockdown, followed by a change in slope to an upward trend, whereby the proportion of new users who were at risk of alcohol dependence increased after the first lockdown in the United Kingdom, though the magnitude of this trend appeared small (Table 4).

We did not detect a significant trend over the study period or a step change or change in slope following the introduction of the first UK lockdown in age or AUDIT scores of new app users (Table 4).

**Table 4:** Results of the best-fitting model for each outcome variable for RQ3a^a^ (N=440 days; range 1-245 users per day).

Outcome variables		B (95% CI)	*P* value
**Gender^b,c^, linear model**			
	Trend	–0.0001 (–0.0003 to 0.0001)	.10
	Level	0.0109 (–0.0692 to 0.0910)	.79
	Slope	0.0013 (0.0001-0.0025)	.04
**Age^b,c^, linear model**			
	Trend	0.0006 (–0.0036 to 0.0048)	.77
	Level	0.3648 (–1.5329 to 2.2625)	.71
	Slope	0.0015 (–0.0272 to 0.0302)	.92
**Employment type^b^, linear model**			
	Trend	0.0000 (–0.0001 to 0.0001)	.65
	Level	0.0367 (–0.0271 to 0.1005)	.26
	Slope	–0.0010 (–0.0020 to 0.0000)	.04
**AUDIT^d^ score^b^, linear model**			
	Trend	0.0020 (–0.0003 to 0.0043)	.09
	Level	–0.3994 (–1.4571 to 0.6583)	.46
	Slope	0.0044 (–0.0116 to 0.0204)	.59
**At risk of alcohol dependence^b,c^, quadratic model**			
	Trend	0.0000 (–0.0001 to 0.0001)	.73
	Level	–0.0596 (–0.1060 to –0.0132)	.01
	Slope	0.0028 (0.0009-0.0047)	.004
	Slope^2^	0.0000 (0.0000-0.0000)	.01

^a^RQ: research question.

^b^Adjusted for month of the year (cubic spline) and day of the week (cubic spline).

^c^Adjusted for autocorrelation.

^d^AUDIT: Alcohol Use Disorders Identification Test.

### Effect of the First UK Lockdown on Engagement With the Drink Less App Among New Users (RQ3b-g)

There was no significant trend in the number of days used by new users across the study period and no change in slope. However, there was a step increase in the number of days used by new users immediately following the introduction of the first UK lockdown (Table 5).

**Figure 4:**
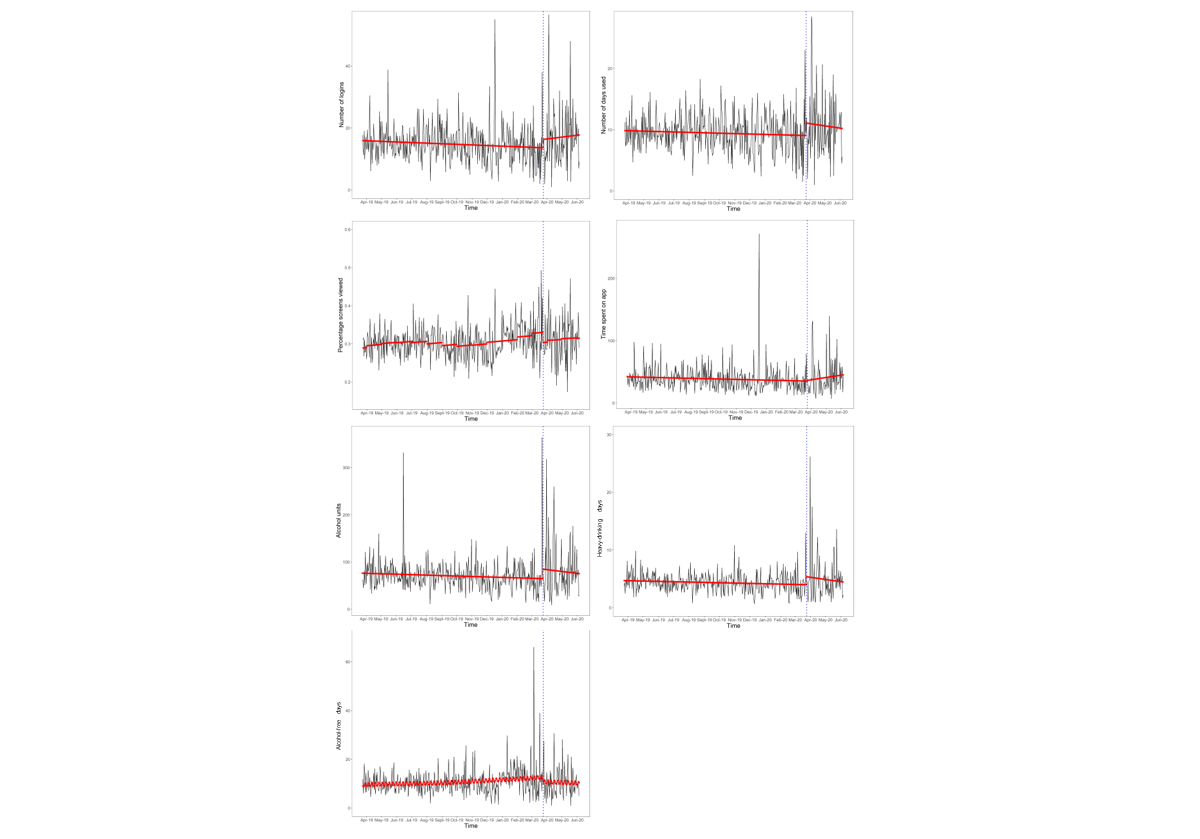
Aggregated engagement indicators among new users of the Drink Less app over the study period (RQ3b-e). The red line indicates fitted values, the gray area indicates the 95% CI, and the dashed blue line indicates the interruption. RQ: research question. Higher-resolution version of this figure is available in Multimedia Appendix 1.

There was an overall upward trend in terms of the percentage of available screens viewed across the study period by new users. There was a step decrease immediately following the introduction of the first UK lockdown, and the upward trend stabilized, with no significant trend during the lockdown (Table 5 and Figure 4).

There were no significant trends across the whole study period for mean alcohol units or heavy-drinking days reported by new users. However, there was a step increase for both following the introduction of the first UK lockdown but no significant change in slope.

There was an overall upward trend in alcohol-free days reported over the whole study period by new users, with no significant step change. The upward trend stabilized, with no significant trend during the lockdown.

There was no overall trend in the number of logins or time spent on the app in minutes across the whole study period for new users, with no significant step change and no significant change in slope following the introduction of the first UK lockdown.

**Table 5:** Results of the best-fitting model for each outcome variable for RQ3b-g^a^ (N=440 days; range 1-245 users per day).

Outcome variables			B (95% CI)	*P* value
**Number of logins^b^, linear model**				
	Trend	–0.0063 (–0.0126 to 0.0000)		0.05
	Level	2.6500 (–0.6376 to 5.9376)		0.12
	Slope	0.0262 (–0.0437 to 0.0961)		0.46
**Number of days used^b^, linear model**				
	Trend	–0.0022 (–0.0055 to 0.0011)		0.19
	Level	2.0484 (0.3449-3.7519)		0.02
	Slope	–0.0099 (–0.0461 to 0.0263)		0.59
**Percentage screens viewed^b^, linear model**				
	Trend	0.0001 (0.0000-0.0002)		0.00
	Level	–0.0265 (–0.0511 to –0.0019)		0.04
	Slope	0.0000 (–0.0005 to 0.0005)		0.87
**Time spent on app (minutes)^b,c^, linear model**				
	Trend	–0.0188 (–0.0408 to 0.0032)		0.09
	Level	0.9802 (–10.4248 to 12.3852)		0.87
	Slope	0.1412 (–0.1013 to 0.3837)		0.25
**Alcohol units^b^, linear model**				
	Trend	–0.0314 (–0.0668 to 0.0040)		0.08
	Level	20.1247 (1.7605-38.4889)		0.03
	Slope	–0.0975 (–0.4880 to 0.2930)		0.62
**Heavy-drinking days^b,c^, linear model**				
	Trend	–0.0019 (–0.0039 to 0.0001)		0.06
	Level	1.3845 (0.3318-2.4372)		0.01
	Slope	–0.0105 (–0.0329 to 0.0119)		0.36
**Alcohol-free days^b,c^, linear model**				
	Trend	0.0083 (0.0028-0.0138)		0.003
	Level	–1.7637 (–4.3836 to 0.8562)		0.19
	Slope	–0.0144 (–0.0692 to 0.0404)		0.61

^a^RQ: research question.

^b^Adjusted for month of the year (cubic spline) and day of the week (cubic spline).

^c^Adjusted for autocorrelation.

## Discussion

### Principal Findings

Following the first COVID-19 lockdown in the United Kingdom, there was a significant increase in the time spent on the app and in the mean alcohol units per day and the number of heavy-drinking days recorded by existing, regular users of the Drink Less app, although no change was detected in the percentage of screens viewed, the number of sessions logged, or in the number of alcohol-free days recorded. There was no increase in downloads per day following the lockdown, although the overall negative trend in new daily app downloads plateaued following the introduction of the first UK lockdown. Among the new users of Drink Less, there were increases in the proportion of female users, manual workers, and those at risk of alcohol dependence following the first UK lockdown. With regard to changes in engagement indicators, there was a step increase in the number of days the app was used but a step decrease in the percentage of available screens viewed within Drink Less, suggesting that users engaged with the app for a longer period but with less of the available content. In terms of drinking characteristics, new users reported step increases in the mean number of alcohol units and heavy-drinking days aggregated over 28 days after app download following the first UK lockdown.

### Strengths and Limitations

A strength of this study is that it was a natural experiment based on longitudinal data exploring self-motivated engagement with a freely available alcohol reduction app in the real world in a large sample against the backdrop of a global pandemic. However, there are also limitations associated with this approach. The period here reflects the immediate effects of COVID-19, which may not have been consistent over a longer period. These findings were also isolated to the United Kingdom. The app is reliant on self-reported alcohol consumption data. It is possible that changes in drinking contexts during the lockdowns could have affected the accuracy of self-report data. People were likely to be drinking in smaller groups in private, rather than public, settings, where they may have been more willing or less likely to forget to log drinks. Conversely, logged drinks during the lockdown might be more likely to be underestimated as individuals pouring their own drinks at home may be less likely to use standard measures than in on-trade settings. Finally, although the GAMMs incorporated cyclic cubic terms for day and month, additional seasonality terms may have further improved the model fit. This should be explored in future research involving app data. A recent study showed that COVID-19 had different effects on health behaviors in different countries, increases in alcohol consumption during the early months of the pandemic were recorded in the United Kingdom and Ireland, and decreases in consumption were recorded among 20 other European countries within the same period [36]. As such, these findings are unlikely to be generalizable to other countries. Furthermore, we were unable to account for differences in living situations, such as whether an individual was furloughed or a parent, which have both been shown to be associated with changes in drinking in lockdown [6].

### Avenues for Future Research

This research focused on 1 digital intervention, and it would be of interest to attempt to triangulate these findings across other forms of digital support available in the United Kingdom and internationally. This would aid in building a comprehensive overview of the effect of the COVID-19 pandemic on the use of digital alcohol reduction support and drinking patterns. The longer-term impact of the ongoing pandemic on engagement with digital interventions is also of interest. Furthermore, although increased engagement is positive, it is also necessary to examine the success of alcohol reduction attempts and whether they can be better supported.

### Implications for Policy and Practice

This study indicated increases in units of alcohol consumed and heavy-drinking days among both existing and new users of the Drink Less app following the first UK lockdown. This is in line with other research outlining the polarizing impact of the first UK lockdown on alcohol consumption [6,12], with increases in consumption seen in increasing and higher-risk drinkers [4] and increases in the frequency of heavy episodic drinking [6]. There was no change in alcohol-free days recorded, suggesting increases in the amount consumed but not the frequency of drinking. Alongside this, there was some indication of increased engagement, with increases in the time spent on the app among existing users and increases in the number of days used but declines in the number of screens used among new users. This supports research outlining increases in self-reported reduction attempts by high-risk drinkers [4]. Increased engagement with the Drink Less app could have been partly due to reductions in the availability of health care services throughout the first lockdown, though this is speculative.

There were also increases in the proportion of female users, manual workers, and those at risk of alcohol dependence following the first UK lockdown. Shifts in engagement with the Drink Less app may be linked to more dramatic changes in lifestyles throughout the lockdown. There is evidence that women were disproportionately affected by additional caring responsibilities during the lockdowns [34,35] and reported greater reductions in well-being [29,37]. Those working in manual professions may have been more likely to have been furloughed than those working in office jobs who could work remotely, which could have resulted in more time to engage with the Drink Less app. Increases in engagement among those at risk of alcohol dependence could be particularly promising as heavier drinking is associated with increased risks of harm [1]. Increases in the proportion of heavier drinkers during the pandemic has persisted in the longer-term postlockdown in the United Kingdom, resulting in significantly increased health and economic burden in England [38,39]. As such, it is important to capitalize on increased interest in alcohol reduction by increasing funding and resources for alcohol support services. Alcohol reduction campaigns specifically targeted at those who have increased their consumption throughout the pandemic may also be useful.

### Conclusion

Following the first COVID-19 lockdown in the United Kingdom, there is some evidence of increased engagement with the alcohol reduction app Drink Less*,* the previously negative trend in new downloads plateaued, and there was an increase in the time spent on the app among regular, existing users and a step increase in the number of days used among new users during the first lockdown. However, there was also evidence of increased alcohol consumption in the first lockdown, with increases in units consumed and heavy-drinking days among existing users and step increases in units consumed and heavy-drinking days among new users.

## Appendix

Multimedia Appendix 1 Higher resolution versions of Figure 1-Figure 4.

## Abbreviations

ACF: autocorrelation function
AIC: Akaike information criterion
AR: autoregressive
AUDIT: Alcohol Use Disorders Identification Test
GAMM: generalized additive mixed model
MA: moving average
PACF: partial autocorrelation function
RQ: research question
