# Supplementary material for: The Effect of the First UK COVID-19 Lockdown on Users of the Drink Less App: Interrupted Time Series Analysis of Sociodemographic Characteristics, Engagement, and Alcohol Reduction
Source: J Med Internet Res. 2022 Nov 10;24(11):e42320. doi: 10.2196/42320 (PMC9693718; doi:10.2196/42320)
Supplement: Multimedia Appendix 1 [file jmir_v24i11e42320_app1.docx]

**Multimedia Appendix 1. Higher resolution versions of Figure 1-Figure 4.**

*
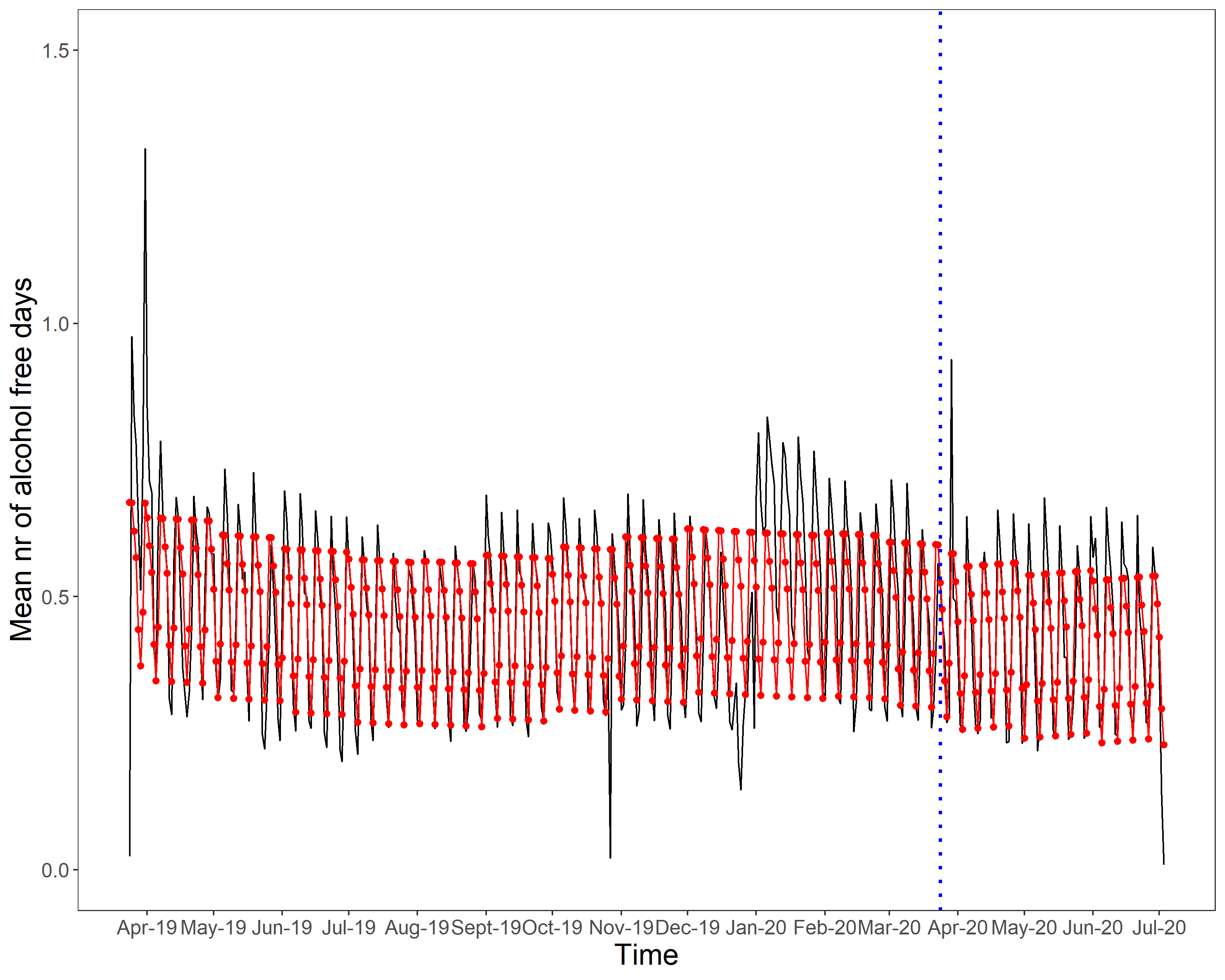

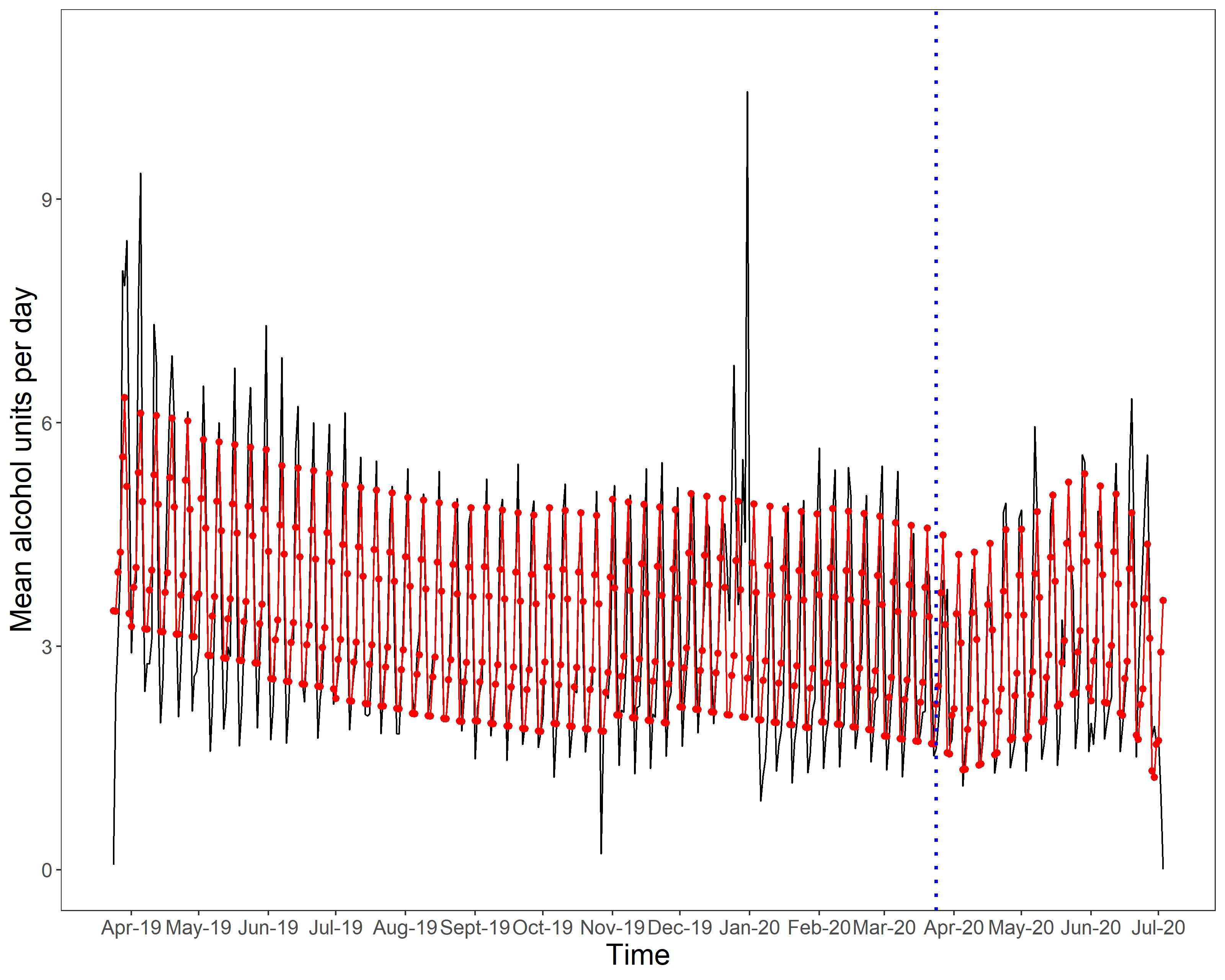

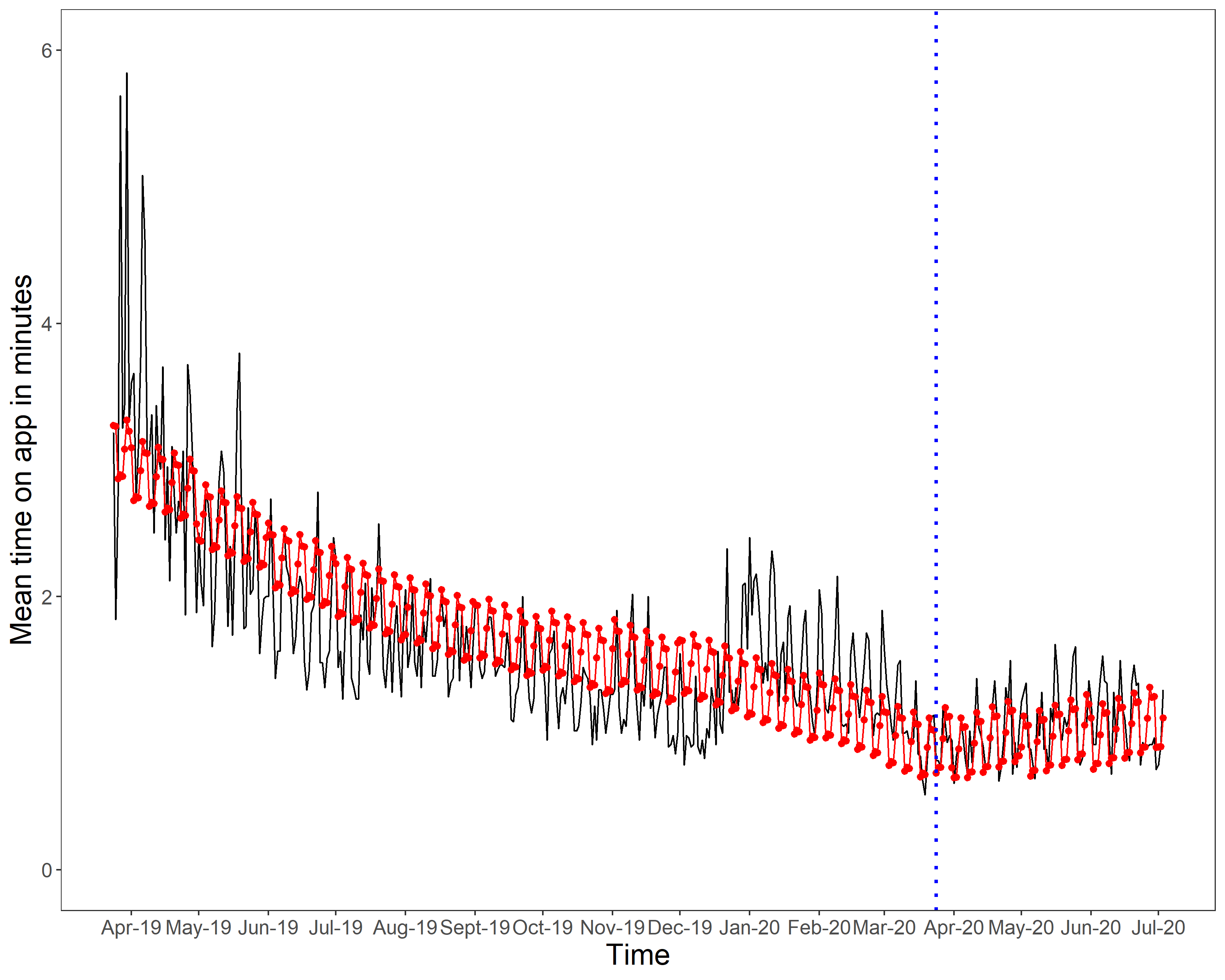

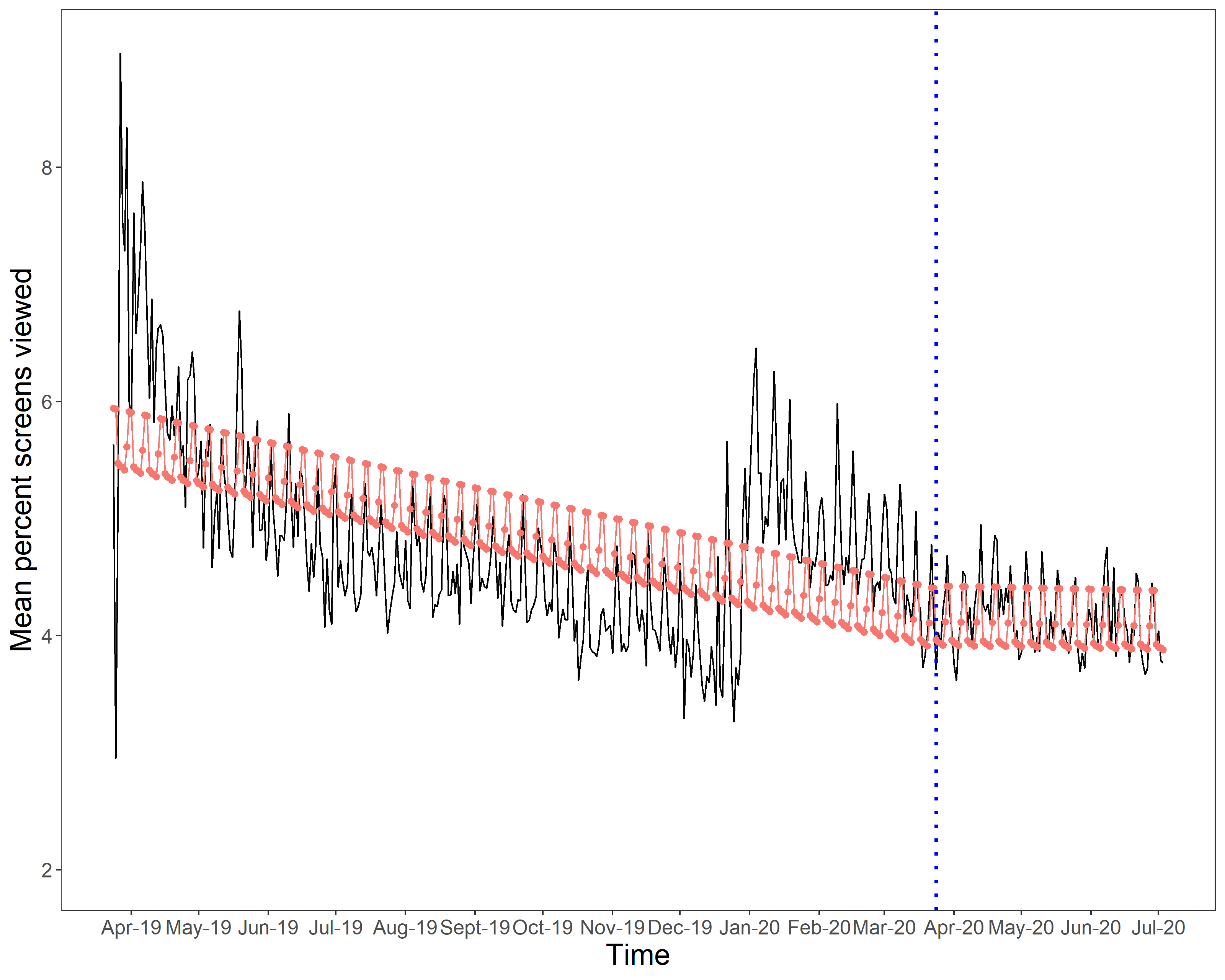

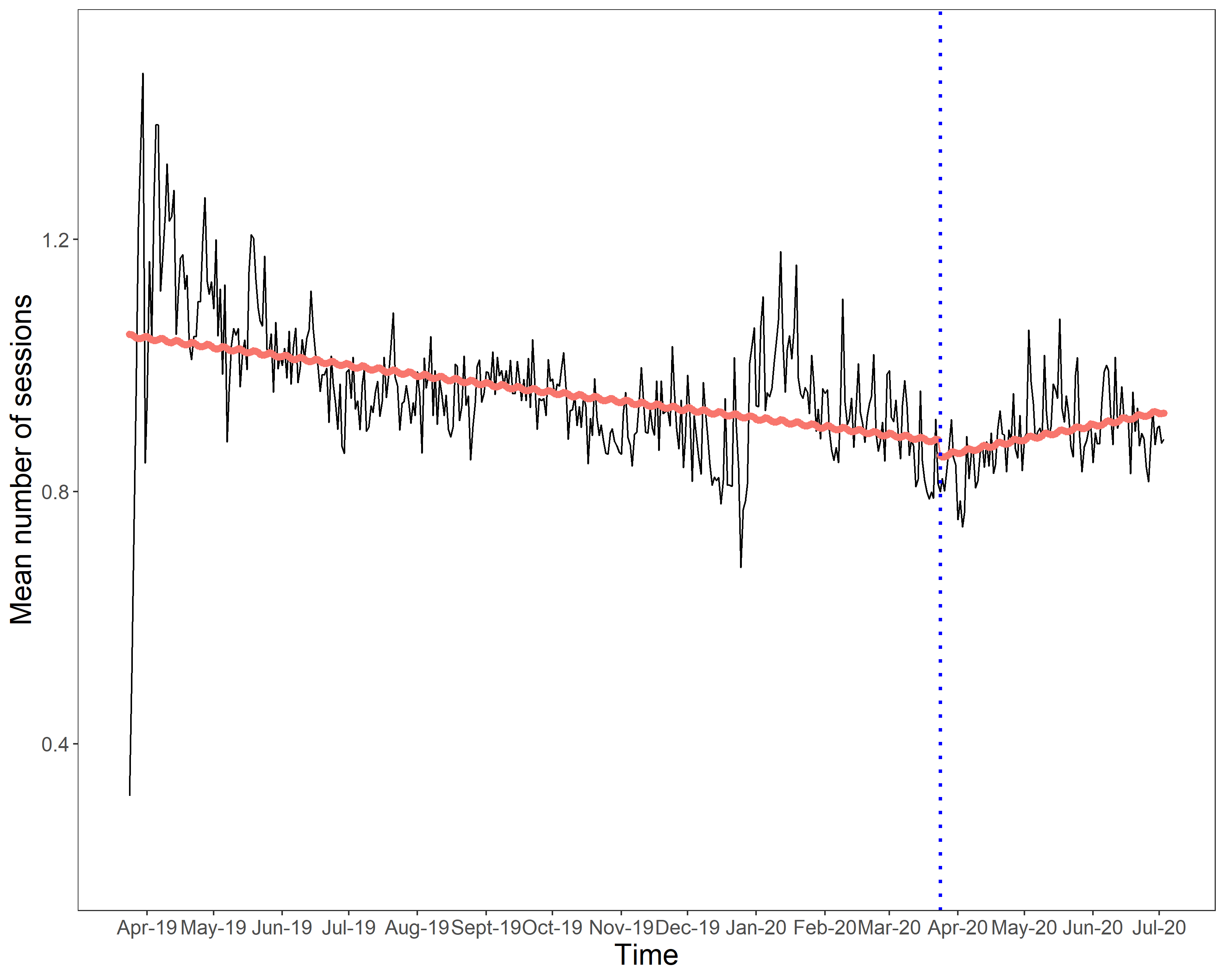
*

Mean percentage of screens viewed

Mean time on app in minutes

*
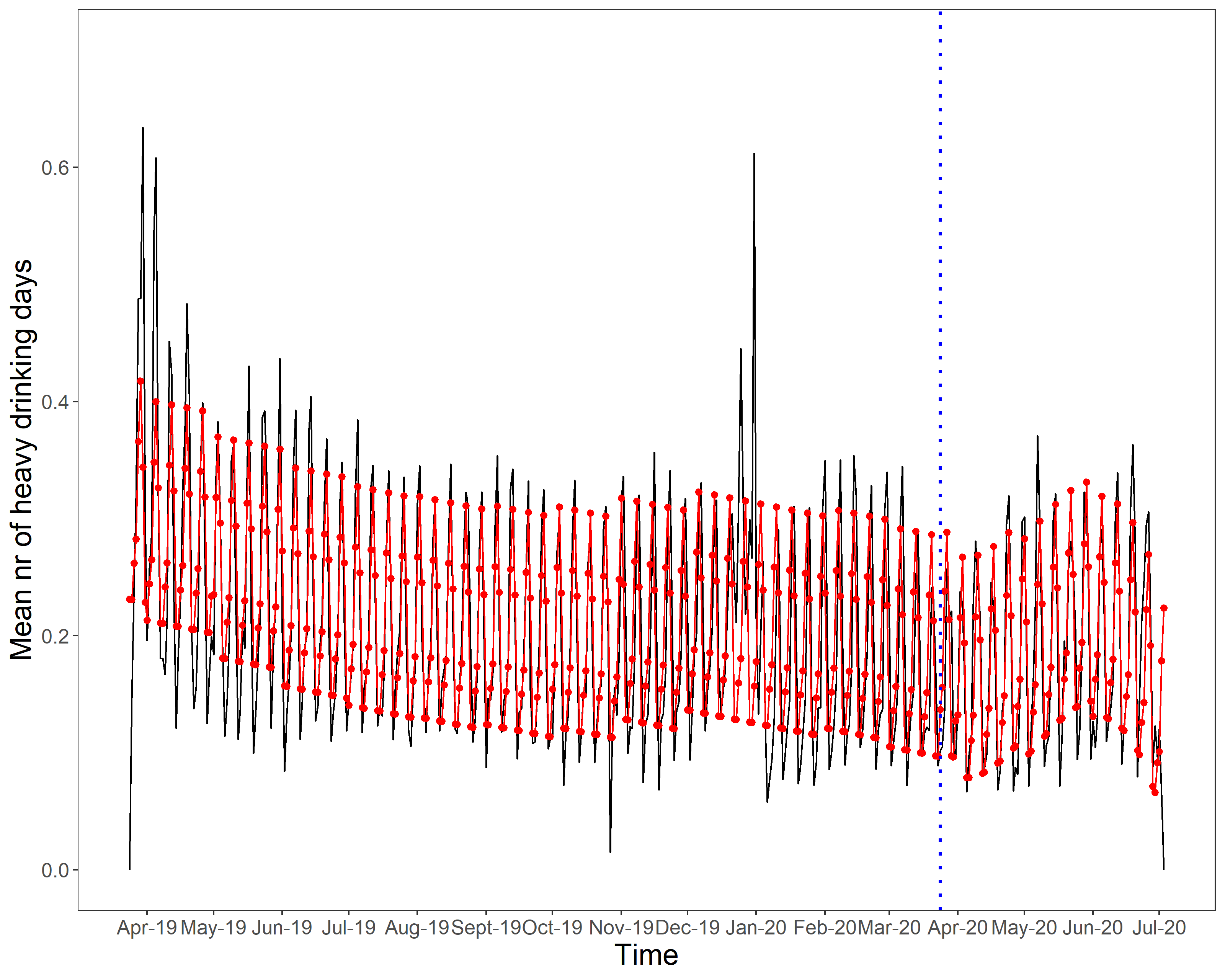
*

Mean alcohol units per day

Mean number of heavy-drinking days

Mean number of alcohol-free days

Mean number of sessions

*Figure 1:* Engagement indicators among existing, regular users of the Drink Less app over the study period (RQ1a-f)*.* The red line indicates fitted values, the grey area 95% CI and dashed blue line indicates the interruption (i.e., the first national UK lockdown).

*
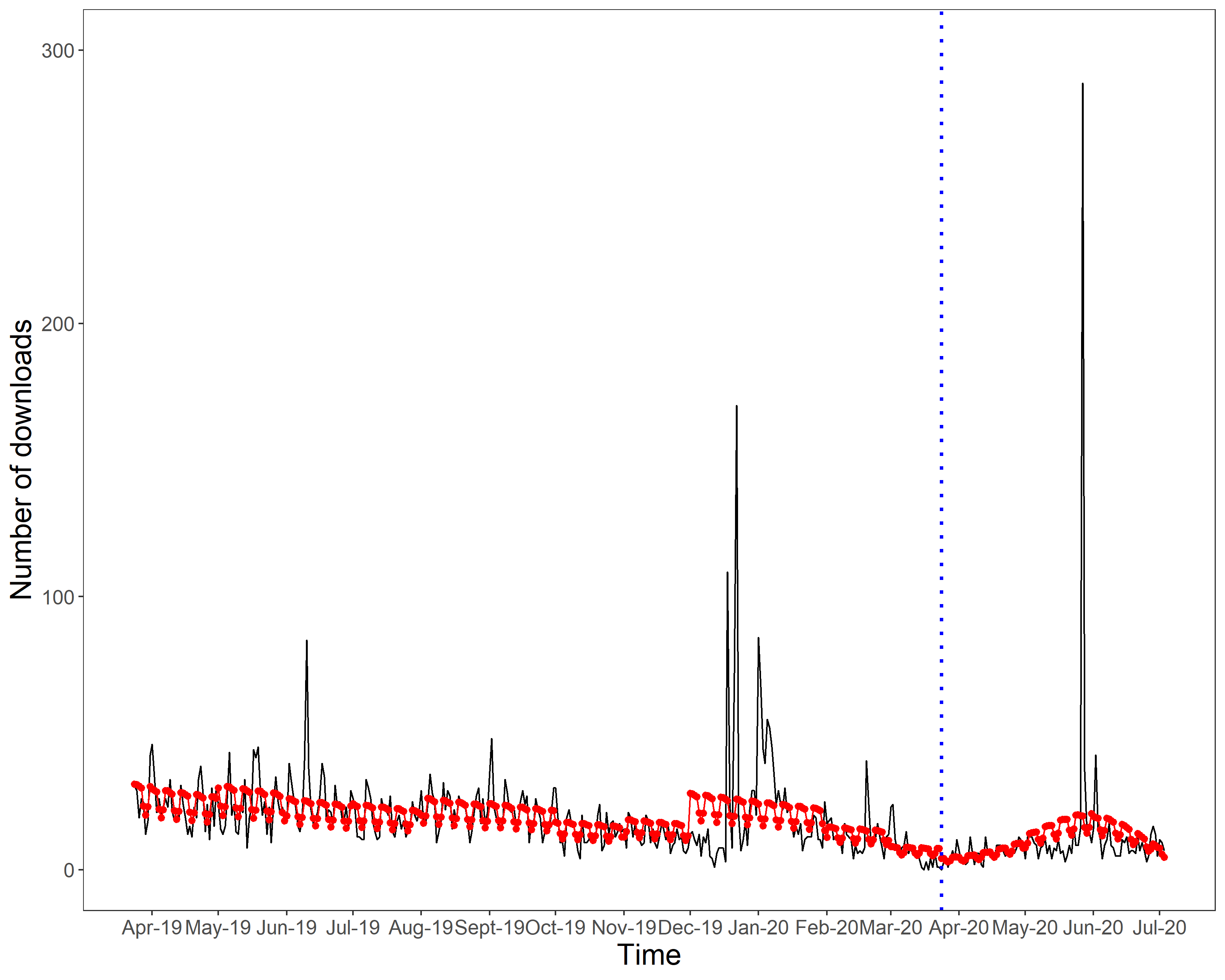
*

*Figure 2:* Number of new *Drink Less* downloads per day over the study period (RQ2). The red line indicates fitted values, the grey area 95% CI and dashed blue line indicates the interruption.


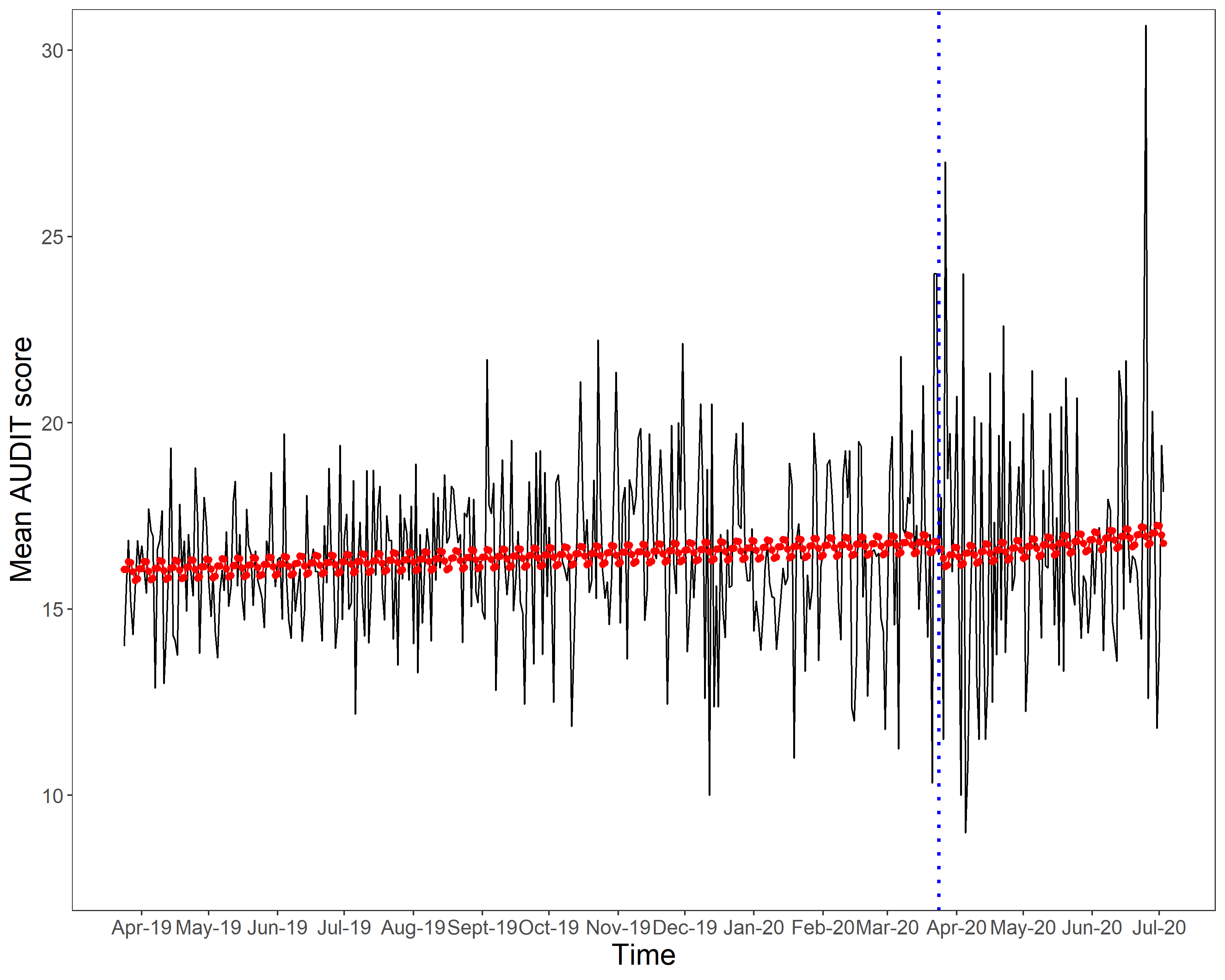

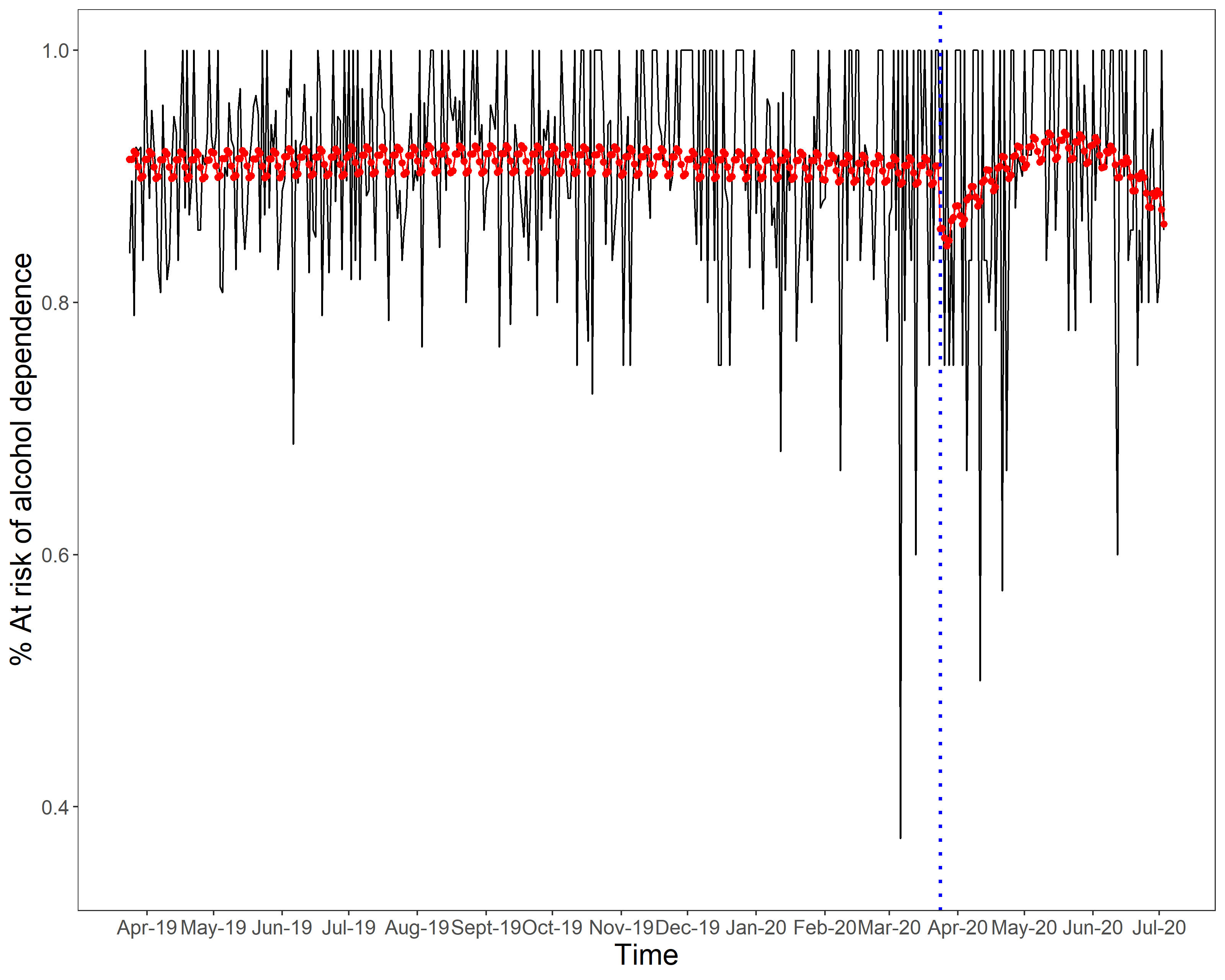

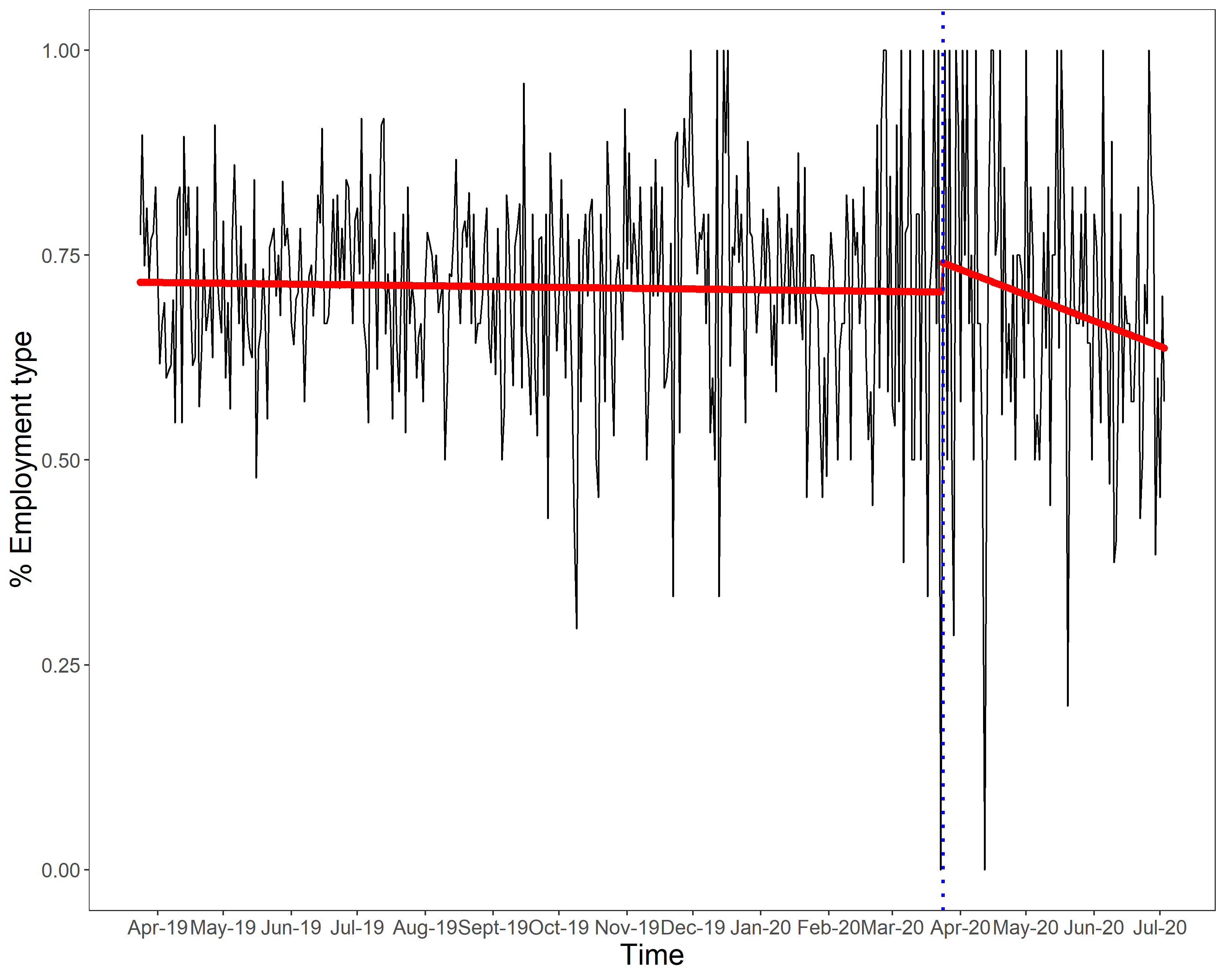

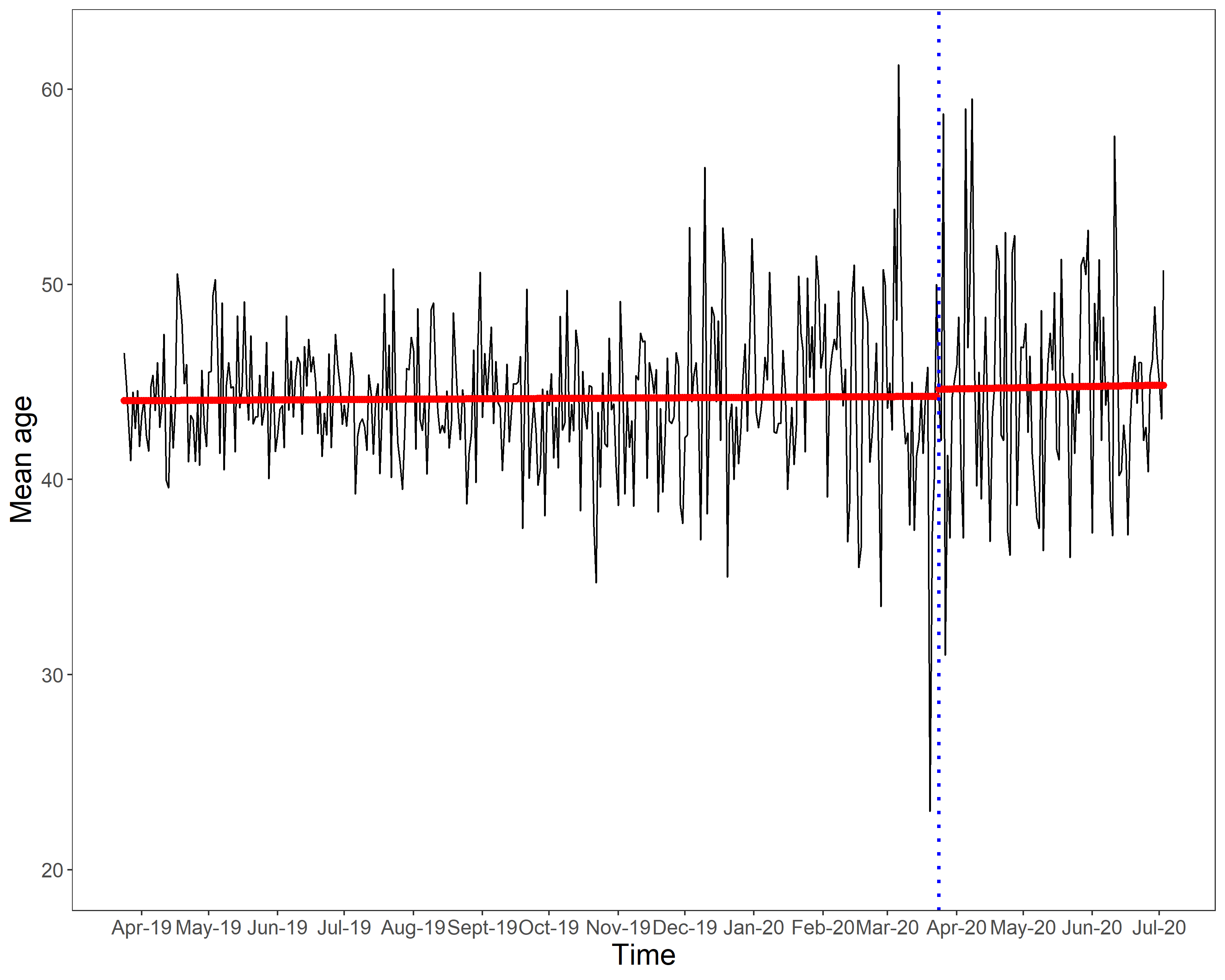

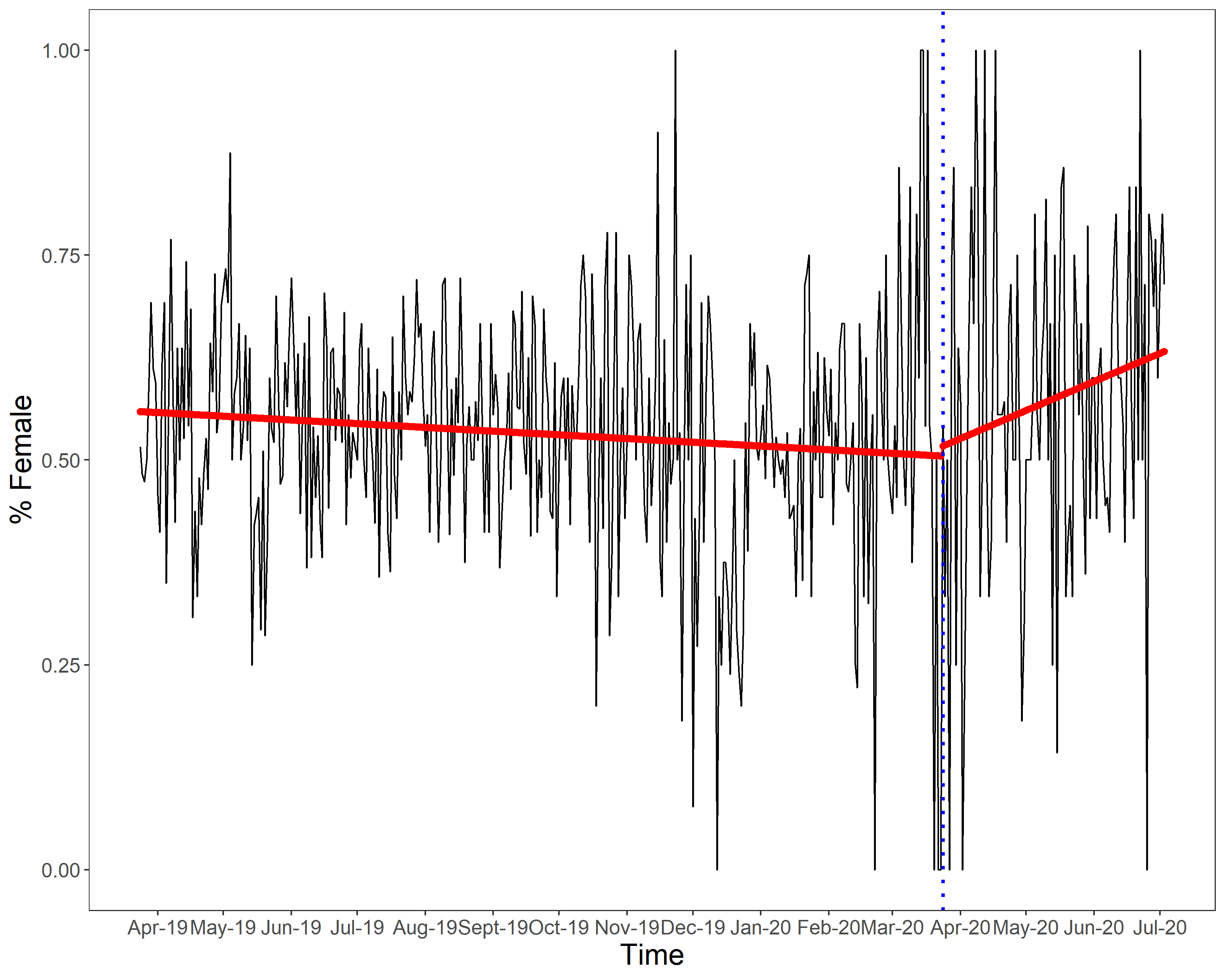


*Figure 3:* Socio-demographic and drinking characteristics of new users of the Drink Less app over the study period (RQ3a). The red line indicates fitted values, the grey area 95% CI and dashed blue line indicates interruption.


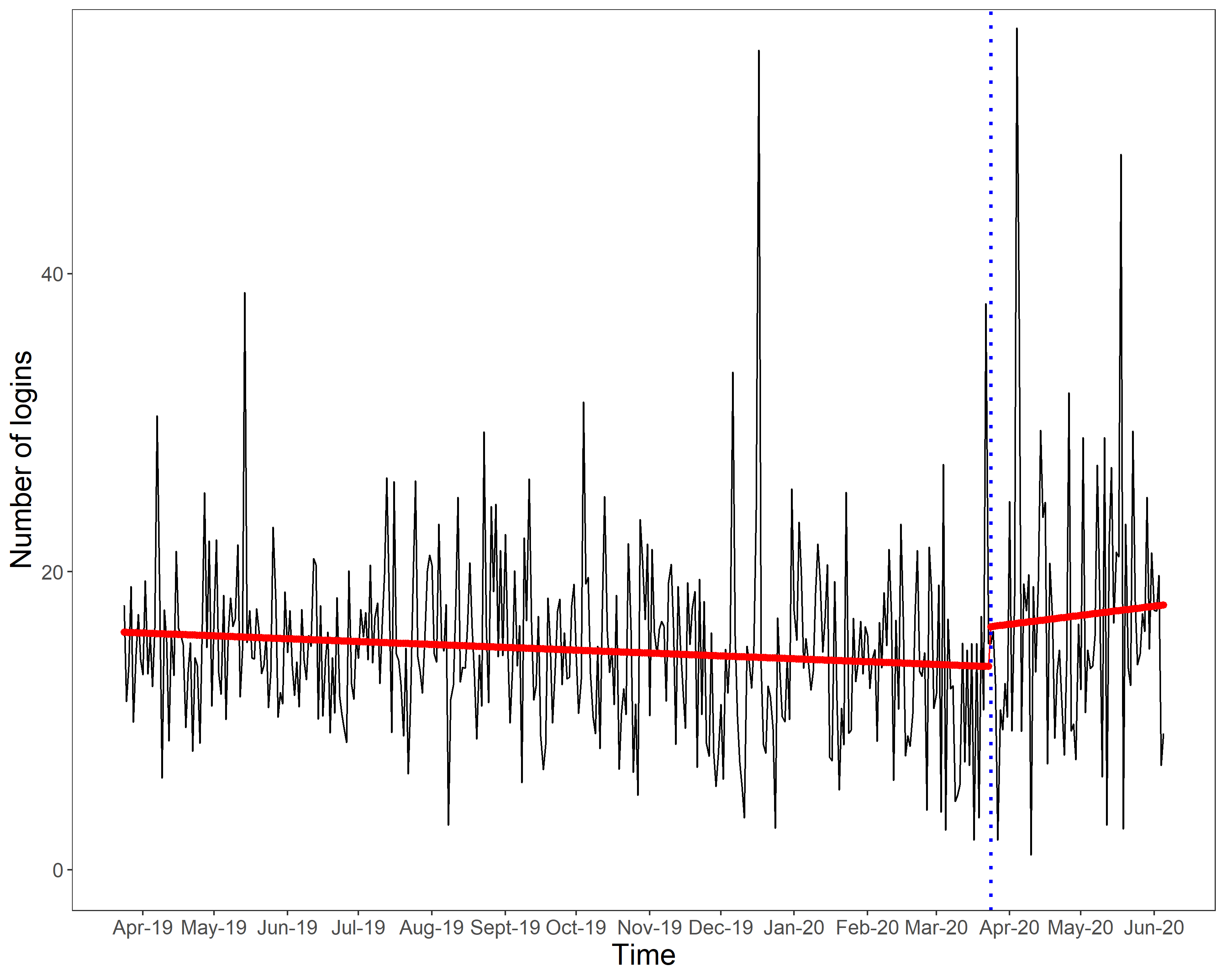

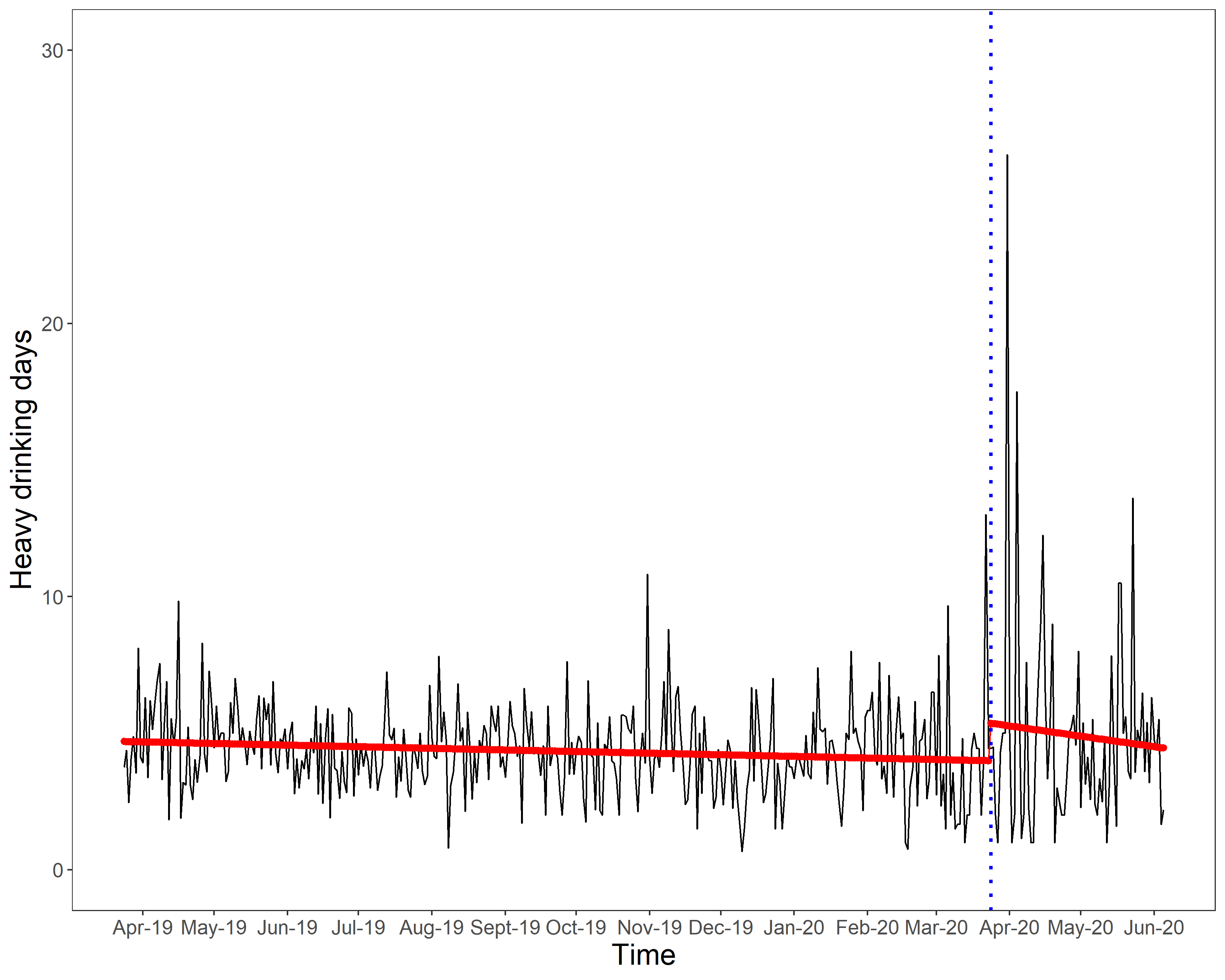

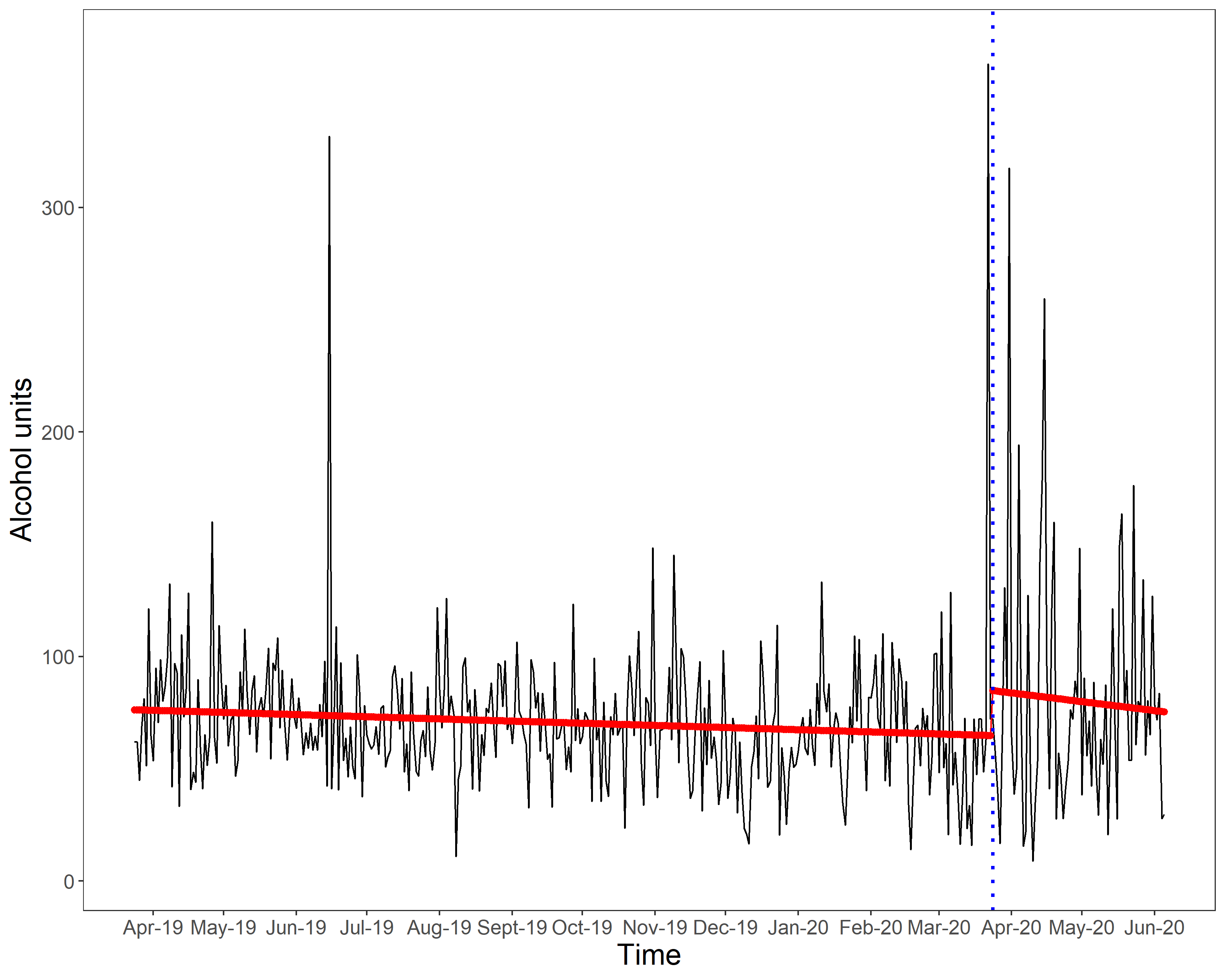

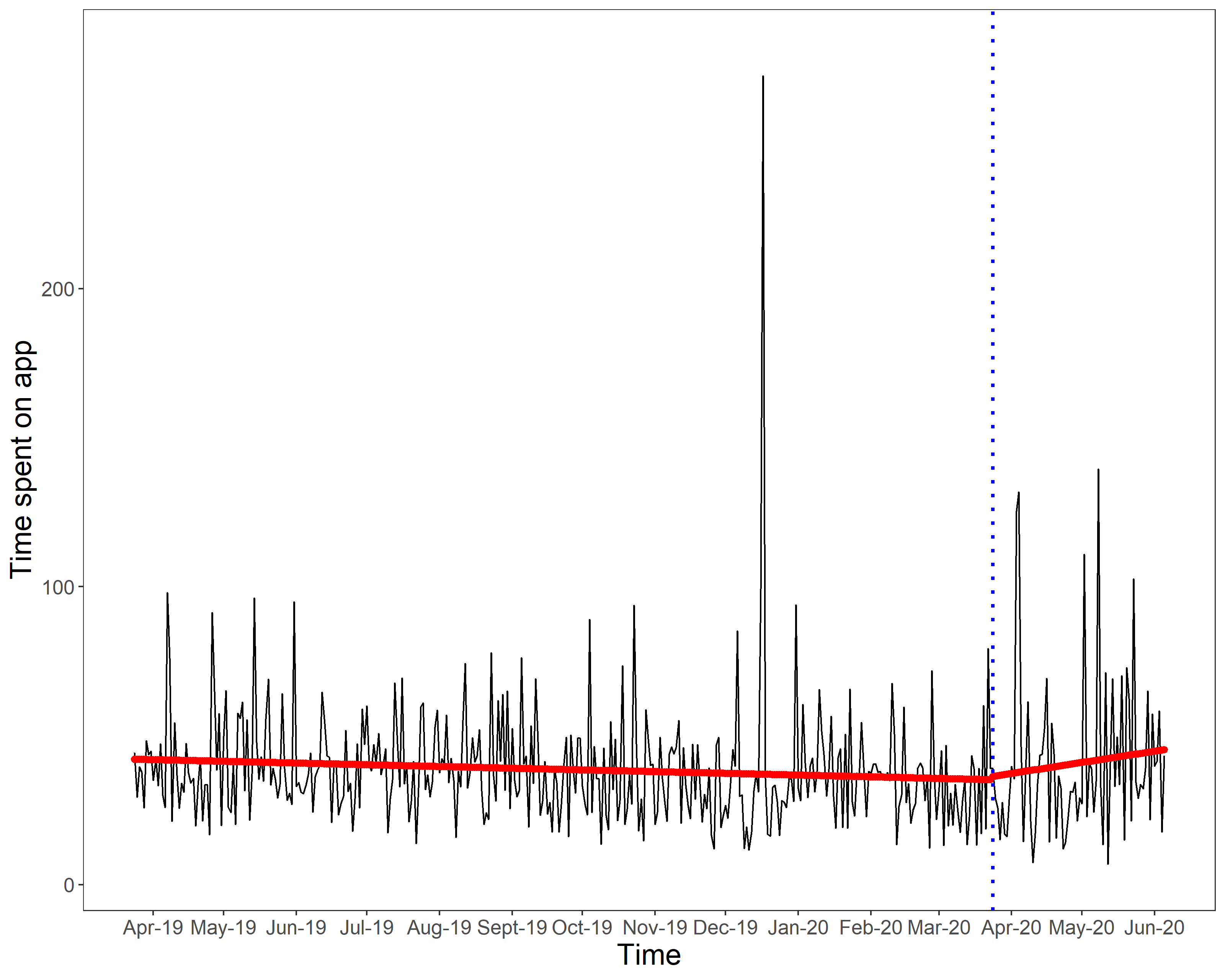

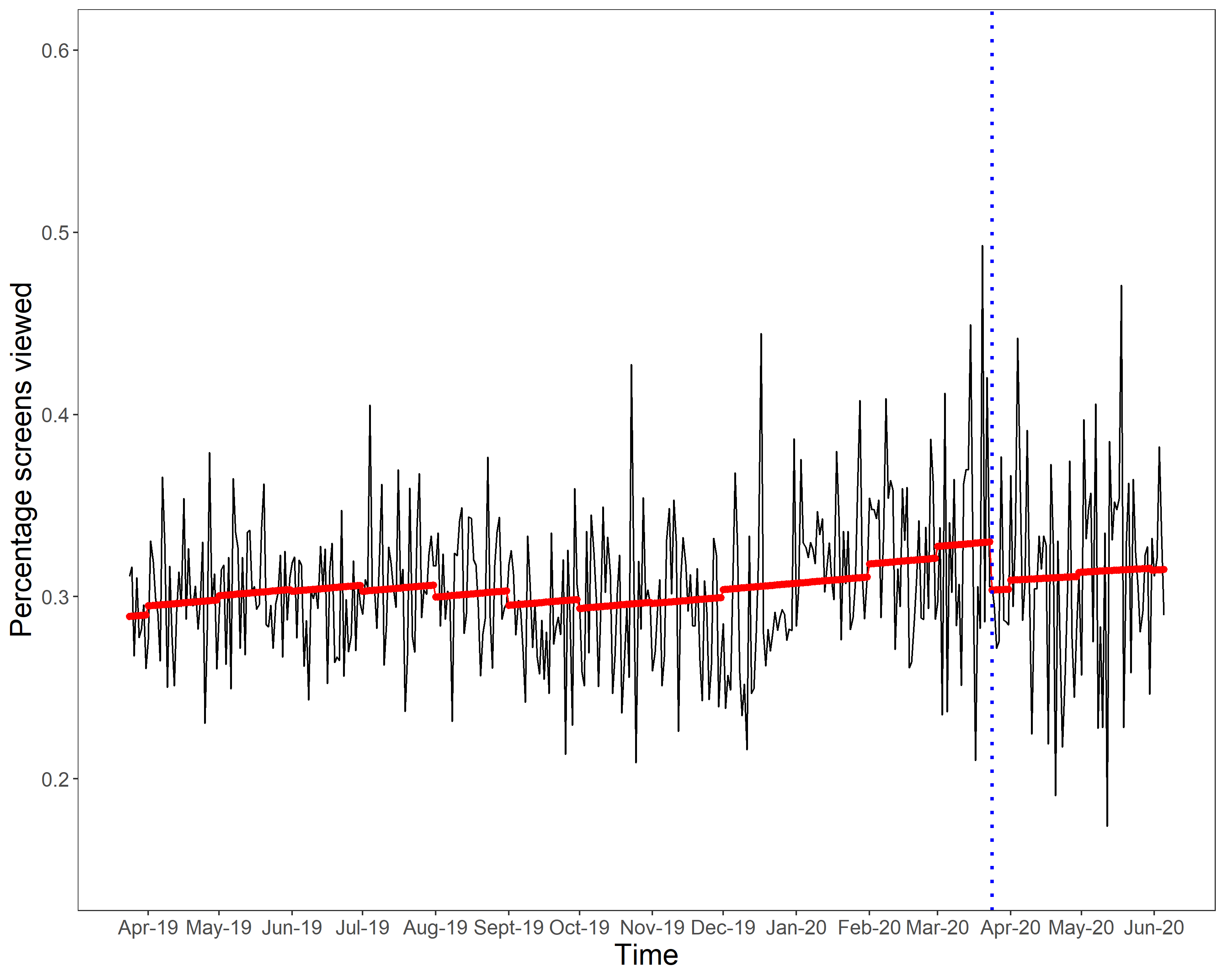

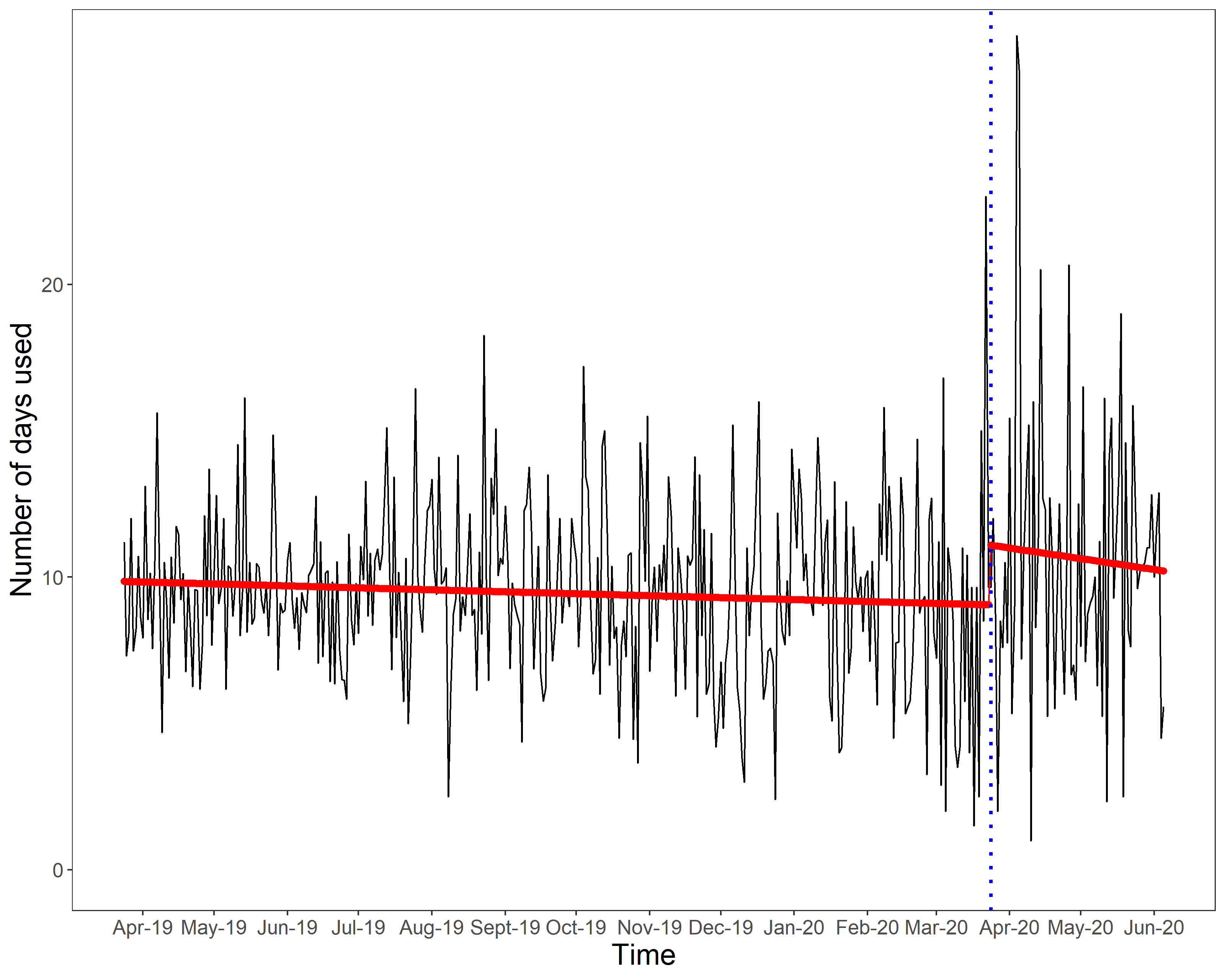


Heavy-drinking days


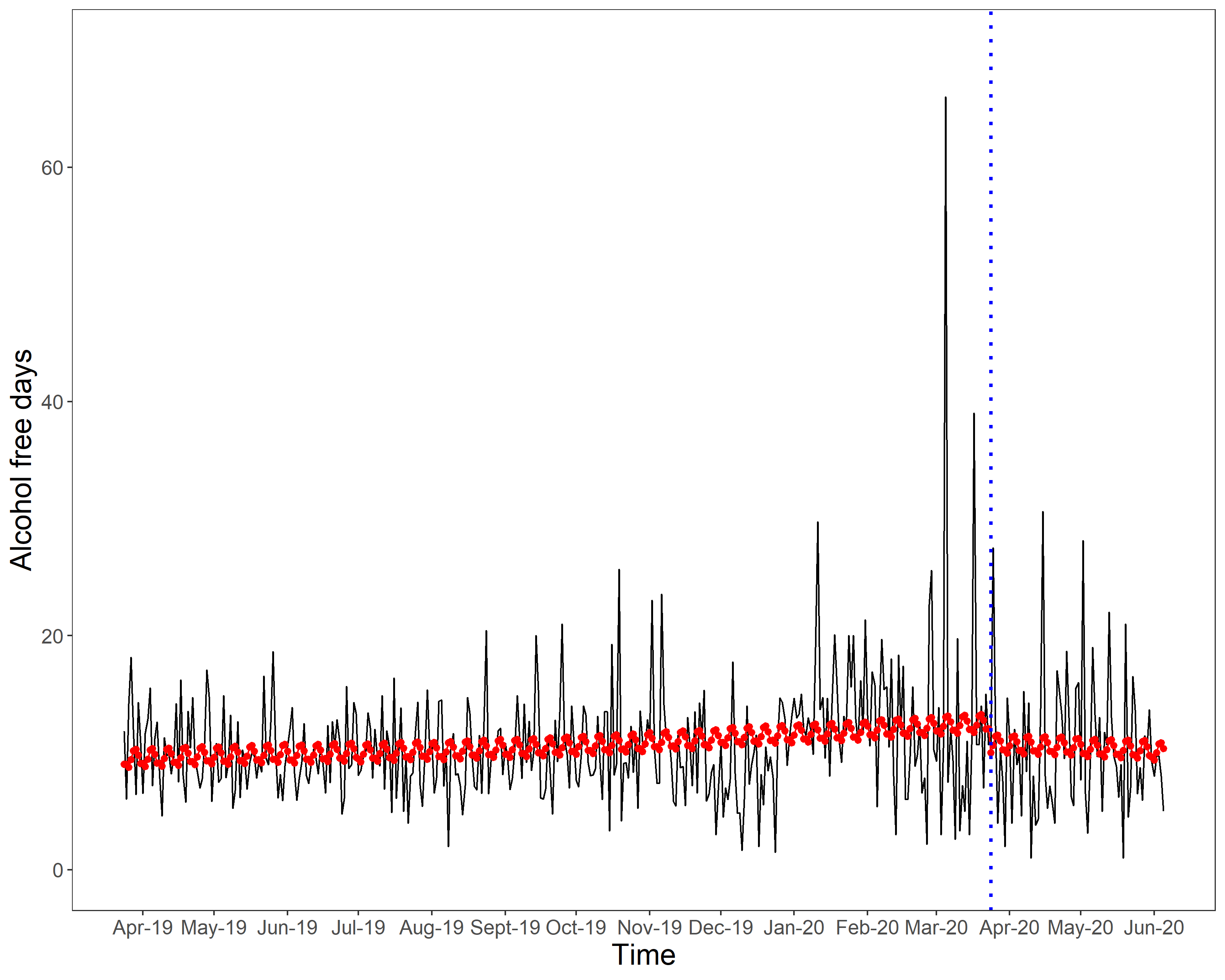


Alcohol-free days

*Figure 4.* Aggregated engagement indicators among new users of the *Drink Less* app over the study period (RQs 3b-e). The red line indicates fitted values, the grey area 95% CI and dashed blue line indicates the interruption.
